# Renal denervation attenuates cardiac dysfunction in HFpEF by inhibiting the ATP-P2X7-NLRP3 inflammasome axis

**DOI:** 10.1007/s00395-025-01138-5

**Published:** 2025-09-16

**Authors:** Zhuqing Li, Xiaoqiang Sun, Yanxin Wang, Feng Zhang, Li Wang, Chunbo Ai, Xu Zhang, Xuemei Yin, Chunlei Liu, Chao Li, Chengzhi Lu

**Affiliations:** 1 Department of Cardiology, Tianjin First Central Hospital, Tianjin, 300192 China; 2 Department of Physiology and Biomedical Engineering, Mayo Clinic, Scottsdale, AZ 85259 USA; 3 First Central Hospital of Tianjin Medical University, Tianjin, 300070 China; 4 School of Medicine, Nankai University, Tianjin, 300071 China

**Keywords:** Heart failure with preserved ejection fraction, Renal denervation, Adenosine triphosphate, P2X7 receptor, NLRP3 inflammasome

## Abstract

Heart failure with preserved ejection fraction (HFpEF) arises from intersecting comorbidities involving inflammation, metabolic stress, and sympathetic nervous system (SNS) activation. To mirror this complexity, we established a clinically oriented 3-hit mouse model combining advanced age, a high-fat diet, and chronic NOS inhibition with NG-Nitro-L-arginine methyl ester (L-NAME). We tested whether renal denervation (RDN) mitigates HFpEF by blunting SNS outflow and the ATP-P2X7-NLRP3 inflammasome axis, and in parallel probed the contribution of this pathway using pharmacologic and cellular approaches. 3-hit mice developed preserved ejection fraction with diastolic dysfunction, cardiomyocyte hypertrophy, interstitial fibrosis, impaired exercise capacity, and elevated inflammatory cytokines, accompanied by increased myocardial ATP, activation of P2X7/NLRP3 signaling, oxidative stress, and pyroptosis.RDN lowered systemic and myocardial norepinephrine, suppressed P2X7-NLRP3 inflammasome activation, reduced fibrosis and cardiomyocyte cross-sectional area, improved E/e′ and exercise capacity, and reduced oxidative stress and myocardial injury. In pharmacological intervention experiments, selective blockade with the P2X7 antagonist A438079 or the NLRP3 inhibitor MCC950 improved diastolic function and exercise capacity and attenuated myocardial fibrosis and cardiomyocyte hypertrophy. Complementary mechanistic studies in cardiomyocytes (H9c2 and primary neonatal rat cardiomyocytes) showed that ATP induced mitochondrial ROS accumulation, inflammasome activation, cytokine release, and cell injury, which were partially reversed by P2X7 knockdown or pharmacological antagonism. Together, these data validate a multi-hit HFpEF model and implicate an SNS-driven ATP-P2X7-NLRP3 axis as a potential therapeutic target. Both RDN and selective pathway inhibition improved diastolic function and attenuated ventricular remodeling, and inform future therapeutic strategies for HFpEF characterized by heightened sympathetic tone.

**Supplementary Information:**

The online version contains supplementary material available at 10.1007/s00395-025-01138-5.

## Introduction

Heart failure with preserved ejection fraction (HFpEF) is diagnosed in patients with typical signs and symptoms of heart failure, a left ventricular ejection fraction (LVEF) ≥ 50%, and objective evidence of elevated left-ventricular filling pressures [24, 37]. HFpEF is a major global health burden with substantial morbidity and mortality, accounting for > 50% of contemporary heart failure hospitalizations and projected to become the predominant subtype as populations age [3, 43]. Despite its rising incidence, patients with HFpEF have few effective pharmacological options, as interventions successful in heart failure with reduced ejection fraction (HFrEF) have shown limited benefit in HFpEF [46]. This therapeutic gap reflects the heterogeneous pathophysiology of HFpEF, which includes cardiomyocyte dysfunction and systemic inflammation, metabolic abnormalities, and vascular remodeling [34, 54].

A significant barrier to advancing HFpEF therapies is the limited availability of animal models that faithfully recapitulate its multifactorial pathophysiology, given the growing recognition that HFpEF arises from systemic dysregulation rather than isolated cardiomyocyte dysfunction [9, 55]. Extending the 2-hit model of high-fat diet (HFD) and N^G^-Nitro-L-arginine methyl ester (L-NAME)–induced hypertension described by Schiattarella et al. [47], a 3-hit paradigm that also incorporates aging may better recapitulate the clinical spectrum of HFpEF. By integrating multiple risk factors, it reproduces metabolic dysregulation, inflammatory activation, and cardiac structural remodeling, thereby supporting more informative mechanistic interrogation and therapeutic evaluation.

Chronic inflammation is a key driver of HFpEF pathophysiology, contributing to myocardial fibrosis and direct cardiomyocyte injury [10]. The NLR family pyrin domain containing 3 (NLRP3) inflammasome is particularly relevant to HFpEF because it mediates caspase-1 activation and the maturation and release of interleukin-1β (IL-1β), a pro-inflammatory cytokine that exacerbates diastolic dysfunction [7]. NLRP3 activation is commonly triggered by extracellular adenosine triphosphate (ATP) acting through P2X purinoceptor 7 (P2X7), leading to K^+^ efflux, Ca^2+^ influx, mitochondrial dysfunction, and increased mitochondrial reactive oxygen species (mtROS) generation [4, 17].

Accumulating evidence indicates that sympathetic nervous system (SNS) overactivation is closely associated with heart failure progression, largely due to its direct and indirect pro-inflammatory effects [22, 27]. In the heart, excessive SNS signaling can enhance ATP release from cardiac sympathetic nerve terminals, thereby activating the P2X7 receptor and the NLRP3 inflammasome and amplifying inflammatory injury, particularly during pressure-overload–induced hypertrophy [49]. Renal denervation (RDN), which interrupts renal sympathetic efferent and afferent signaling, has emerged as a strategy to reduce sympathetic overactivity in conditions such as resistant hypertension and metabolic syndrome [6, 39, 41, 50]. Nonetheless, the precise mechanisms by which RDN attenuates inflammasome activation and influences cardiac remodeling under HFpEF conditions remain insufficiently elucidated.

In this study, we established a 3-hit mouse model integrating aging, hypertension, and metabolic stress to recapitulate key clinical features of HFpEF. We then investigated whether RDN alleviates HFpEF by modulating the ATP-P2X7-NLRP3 axis, thus reducing cardiac inflammation, fibrosis, and diastolic dysfunction. Our findings provide mechanistic insights into the neuroinflammatory drivers of HFpEF and support the therapeutic potential of RDN for this increasingly prevalent heart failure subtype.

## Materials and methods

### Animal model and grouping

All animal procedures complied with the Guide for the Care and Use of Laboratory Animals (US National Institutes of Health, 8th edition, 2011 update) and were approved by the Institutional Animal Care and Use Committee of Nankai University (IACUC Approval No. 2023-SYDWLL-000394). All efforts were made to minimize animal suffering and to use the minimum number of animals necessary to obtain reliable results. Adult male C57BL/6 J mice were housed under a 12:12 h light–dark cycle, with food and water available ad libitum. Only adult male mice were used, as previous work indicates that female mice are less susceptible to HFD plus L-NAME treatment [20]. To establish a clinically oriented multi-hit HFpEF model, four baseline groups were studied (n = 6 per group, 24 mice in total): 3-hit (18-month-old mice given HFD plus L-NAME [0.5 g/L in drinking water] for 5 weeks), CTRL (6-month-old, chow), Aged (18-month-old, chow), and 2-hit (6-month-old, HFD + L-NAME). Three independent interventional experiments were performed, each with four groups (n = 6 per group, 72 mice in total). NHF denotes 6-month-old chow-fed controls; HFpEF denotes the 3-hit model. In the RDN experiment, the groups were NHF, HFpEF, HFpEF + RDN, and HFpEF + Sham. The A438079 experiment included NHF + Vehicle, NHF + A438079, HFpEF + Vehicle, and HFpEF + A438079. The MCC950 experiment included NHF + Vehicle, NHF + MCC950, HFpEF + Vehicle, and HFpEF + MCC950. Animals were randomized, investigators were blinded during data acquisition and analysis, and assessment schedules were identical across groups.

### Cell culture and transfection

The rat embryonic cardiomyocyte line, H9c2, was acquired from the Cell Bank of the Chinese Academy of Sciences and was cultured in high-glucose Dulbecco’s Modified Eagle Medium (DMEM, 11,965,084, Gibco, Thermo Fisher Scientific, Waltham, MA, USA) supplemented with 10% fetal bovine serum (FBS; SH3007103, HyClone, Cytiva, Logan, UT, USA) 100 U/ml penicillin, and 100 μg/ml Streptomycin (S8290, Solarbio, Beijing, China). The culture conditions were maintained at 37 °C and 5% CO_2_. H9c2 cells at 60–70% confluence were transfected with P2X7-specific siRNA (sc-514962, Santa Cruz Biotechnology, Dallas, TX, USA) or scrambled siRNA control (sc-37007, Santa Cruz Biotechnology, Dallas, TX, USA) using Lipofectamine RNAiMAX (13778075, Invitrogen, Thermo Fisher Scientific, Waltham, MA, USA), according to the manufacturer’s instructions. After 24 h to allow for knockdown, cells were stimulated with ATP (5 mM, A1852, Sigma-Aldrich, St. Louis, MO, USA) in DMEM medium for 24 h before subsequent assays.

### Primary neonatal cardiomyocyte isolation and treatment

Neonatal Sprague–Dawley rats (3 days old) were used to isolate primary ventricular cardiomyocytes. Briefly, pups were euthanized, and hearts were rapidly excised and rinsed in ice-cold PBS. Ventricles were minced into ~ 1 mm^3^ fragments and enzymatically digested at 37 °C in 0.25% trypsin plus 0.1% collagenase II. Supernatants from successive digestions were collected into growth medium containing 10% FBS to terminate enzymatic activity. Cells were pelleted by gentle centrifugation and resuspended in high-glucose DMEM supplemented with 10% FBS and penicillin–streptomycin. To enrich cardiomyocytes, the suspension was pre-plated on uncoated dishes for 1 h at 37 °C to allow attachment of nonmyocytes; the non-adherent, cardiomyocyte-enriched fraction was then collected and seeded onto culture plates. Cells were maintained at 37 °C in a humidified 5% CO₂ incubator, and the medium was refreshed after 24 h to remove debris. Primary cardiomyocytes were assigned to four groups: Control (no treatment), A438079 alone, ATP alone, and ATP + A438079. All treatments were performed in high-glucose DMEM. A438079 (P2X7 antagonist, HY-15488A, MedChemExpress, Monmouth Junction, NJ, USA) was applied at 10 μM for 1 h before ATP and was maintained throughout the subsequent ATP exposure. ATP was added to a final concentration of 5 mM and cells were incubated for 24 h. After treatments, cells were harvested for RNA extraction or assays as described below.

### Renal denervation

Bilateral RDN was performed at the start of the HFpEF induction period. Under isoflurane anesthesia, a midline abdominal incision exposed the renal arteries. All visible perivascular nerves were carefully stripped, followed by topical application of 10% phenol in ethanol for 5 min. Sham-operated mice underwent identical procedures but without nerve disruption and were instead treated with saline. RDN success was verified by reduced tyrosine hydroxylase (TH) staining and decreased serum norepinephrine content at endpoint.

### Transcriptomic profiling by bulk RNA sequencing

LV tissue and neonatal rat cardiomyocytes (NRCMs) were profiled by poly(A)-selected, strand-specific RNA-seq (NovaSeq 6000, PE150; ~ 30–50 M pairs/sample), with n = 3 biological replicates per condition. Reads were trimmed, aligned with HISAT2 to mm10 or rn6, and summarized with featureCounts. Low-abundance genes were filtered. DESeq2 identified DEGs (FDR < 0.05; |log2FC|> 0.58 where specified). Functional analyses used clusterProfiler; QC included PCA. Data will be deposited to GEO.

### Quantitative proteomics by data-independent acquisition mass spectrometry

Samples underwent urea lysis, reduction/alkylation, tryptic digestion, C18 cleanup, and nanoLC-Orbitrap DIA (m/z 400–1200, variable windows), with n = 3 biological replicates per condition. DIA-NN (directDIA) controlled FDR at 1% (precursor/protein). Proteins were normalized, filtered (≥ 2 unique peptides; ≤ 30% missing), imputed at low intensity, and tested with limma (FDR ≤ 5%). Enrichment and RNA–protein concordance were performed in R. Data will be deposited to PRIDE.

### Echocardiography

Transthoracic echocardiography was performed using a Vevo 2100 system (VisualSonics, Toronto, ON, Canada) equipped with an MS400 30-MHz Linear array transducer. Mice were induced with 2–3% isoflurane in oxygen (1 L/min) and maintained under light anesthesia (0.8–1.2% isoflurane) to keep heart rate ≥ 450 beats/min. Animals were placed supine on a temperature-controlled platform (37 °C) with integrated ECG electrodes; body temperature and ECG were continuously monitored. After chest shaving, pre-warmed ultrasound gel was applied. Cardiac function was assessed using M-mode, pulsed-wave Doppler, and tissue Doppler imaging. Image acquisition and analysis were performed by an operator blinded to group allocation. Scans were immediately terminated if apnea, bradycardia, or other instability occurred, and animals were recovered on a warmed pad.

### Tail cuff blood pressure recordings

Systolic blood pressure was measured noninvasively in conscious mice using the tail-cuff method with a CODA High-Throughput NIBP Controller (CODAHT4, Kent Scientific, Torrington, CT, USA). Animals were positioned in individual holders on a temperature-controlled platform maintained at 37 °C, and recordings were conducted under steady-state conditions. Prior to testing, mice were acclimatized to brief restraint. Blood pressure recordings were taken over a minimum of four consecutive days, with the average derived from at least eight measurements per session.

### Exercise exhaustion test

Following three days of acclimatization to treadmill exercise, an exhaustion test was administered to the experimental groups of mice. The animals ran on a level treadmill (SA101, Shulaibao Biotechnology, Wuhan, China) without incline.The protocol began with a warm-up at a speed of 5 m/min for four minutes, followed by an increase to 14 m/min for two minutes. Subsequently, the speed was increased by 2 m/min every two minutes until exhaustion, which was defined as the animal's inability to resume running within 10 s after direct contact with an electric stimulus grid. Both the running time and distance were recorded and calculated, respectively.

### Histological assessment

Following euthanasia, Hearts were rapidly excised, rinsed in ice-cold PBS, blotted dry, and subdivided. One portion was immersion-fixed in 10% neutral buffered formalin at room temperature for 48 h, processed for paraffin embedding, and sectioned at 5 μm for histology. These sections were deparaffinized, rehydrated by sequential immersion in xylene and alcohol, and subjected to hematoxylin and eosin (H&E) staining. For H&E, sections were stained with Hematoxylin for eight minutes and eosin for one minute, followed by a wash in 0.5% hydrochloric acid in alcohol, dehydration, and mounting. H&E-stained heart sections were also used to quantify right ventricular (RV) free wall thickness as an index of cardiac remodeling. For this measurement, cross-sectional images at the level of the papillary muscles were analyzed using ImageJ software. RV free wall thickness was defined as the distance from the endocardial surface to the epicardial surface in the mid-portion of the RV free wall. Masson’s trichrome staining adhered to the manufacturer’s protocol using a commercially available kit (G1340, Solarbio, Beijing, China). Fibrosis area was quantified as the average percentage of fibrosis across five randomly selected fields per heart. Cardiomyocyte cross-sectional area was determined by staining the sections with Wheat Germ Agglutinin (WGA, Alexa Fluor^TM^488 conjugated; W11261, Invitrogen, Carlsbad, CA, USA) at 5 μg/ml in the dark for ten minutes; nuclei were counterstained with DAPI (1 mg/ml, 9542, Sigma-Aldrich, St. Louis, MO, USA). Sections were examined with a fluorescence microscope (AX10 imager A2/AX10 cam HRC; Carl Zeiss, Oberkochen, Germany) and imaged using ZEN software (Zeiss, Oberkochen, Germany). For each section, 10–15 images were captured from random fields. A minimum of 100 DAPI-positive cardiomyocytes per image were analyzed, and the results were averaged. Image analysis was performed using Image J software (version 1.53c, National Institutes of Health, Bethesda, MD, USA), and representative images for each group were selected based on typical histological or fluorescence features.

### TUNEL staining

Cardiomyocyte apoptosis was evaluated using the TUNEL (terminal deoxynucleotidyl transferase dUTP nick-end labeling) assay. Paraffin-embedded heart Sections (5 μm) were deparaffinized in xylene, rehydrated through a graded ethanol series, and subjected to antigen retrieval in citrate buffer (10 mM, pH 6.0) at 95 °C for 10 min. After cooling, sections were incubated with proteinase K (20 μg/mL) for 15 min at room temperature to permeabilize the tissue. Apoptotic cells were then labeled using a TUNEL assay kit (T2130, Solarbio, Beijing, China) according to the manufacturer’s instructions. Nuclei were counterstained with DAPI. Fluorescent images were acquired using a fluorescence microscope (AX10 imager A2/AX10 cam HRC; Carl Zeiss, Oberkochen, Germany), and TUNEL-positive nuclei were quantified in at least five randomly selected fields per section. The apoptotic index was calculated as the percentage of TUNEL-positive nuclei relative to total DAPI-positive nuclei.

### Transmission electron microscopy

Cardiac tissue samples were fixed in 2.5% glutaraldehyde (G1102, Servicebio, Wuhan, China) at 4 °C for 2–4 h. Post-fixation was performed using 1% osmium tetroxide in 0.1 M phosphate buffer (pH 7.4) at room temperature for 2 h. Samples were then dehydrated through a graded ethanol series (50%, 70%, 80%, 90%, 95%, and 100%) followed by two changes in 100% acetone (Sinopharm Chemical Reagent Co., Ltd., Shanghai, China), each for 15 min. Subsequently, tissues were infiltrated with a mixture of acetone and EMbed-812 resin (90529-77-4, SPI-Chem, West Chester, PA, USA) in ratios of 1:1 for 2–4 h and 1:2 overnight, followed by pure resin infiltration for 5–8 h. Specimens were embedded in pure EMbed-812 resin and polymerized at 60 °C for 48 h. Ultrathin Sections (60–80 nm) were cut using an ultramicrotome (Leica EM UC7, Leica Microsystems, Wetzlar, Germany) equipped with a diamond knife (Ultra 45°, DiATOME, Biel, Switzerland). Sections were stained with 2% uranyl acetate and lead citrate, then examined under a transmission electron microscope (HT7700, Hitachi, Tokyo, Japan) operating at 120 kV. Images were captured using a digital camera system attached to the microscope.

### RNA isolation and qPCR

Total RNA was extracted from murine hearts and cultured cardiomyocytes using TRIzol reagent (15,596,026, Thermo Fisher Scientific, Waltham, MA, USA). For reverse transcription, 500 ng of RNA was utilized with the iScript reagent (1,708,890, Bio-Rad, Hercules, CA, USA). Quantitative PCR reactions were conducted in triplicate using SYBR master mix (1,725,150, Bio-Rad, Hercules, CA, USA), while real-time PCR was performed in duplicate employing TaqMan Gene Expression Assay probes with specific primers for target sequences. The relative quantity of target mRNA was estimated using the 2^−ΔΔCT^ method with GAPDH as normalization controls, and fold changes in mRNA expression levels were calculated relative to controls. The sequences of PCR primers used in this study are presented in Supplementary Data, Table S1.

### Western blot, biochemical and ELISA assay

Total proteins were extracted from mouse tissues and cells using a protein lysis buffer that included a protease inhibitor (PMSF plus cocktail). Equal amounts of protein from each sample were analyzed to determine protein expression via Western blot, as described in reference [30]. Western blot images were scanned and band densities were semi-quantitatively analyzed. Proteins were detected with the following primary antibodies: P2X7 (11144–1-AP, Proteintech, Rosemont, IL, USA); NLRP3 (30109–1-AP, Proteintech, Rosemont, IL, USA); apoptosis-associated speck-like protein containing a CARD (ASC) (30641–1-AP, Proteintech, Rosemont, IL, USA); Cleaved-Caspase1 (89332, Cell Signaling Technology, Danvers, MA, USA); Caspase1 (83383, Cell Signaling Technology, Danvers, MA, USA); catalase (CAT) (21260–1-AP, Proteintech, Rosemont, IL, USA); glutathione peroxidase 4 (GPX4) (67763–1-Ig, Proteintech, Rosemont, IL, USA); superoxide dismutase 2, mitochondrial (SOD2) (24127–1-AP, Proteintech, Rosemont, IL, USA).

The oxidative stress markers including malondialdehyde (MDA) content, superoxide dismutase (SOD) activity, glutathione (GSH) content and total antioxidantcapacity (T-AOC) in mouse serum were detected with the corresponding test kits (Solarbio, China). The lactate dehydrogenase (LDH) activity in mouse blood, myocardial tissues, and cell supernatant was detected using the corresponding assay kits (mbbiology, Nanjing, China).

Mouse serum and myocardial tissue levels of norepinephrine (MBS2600834), epinephrine (MBS700597), neuropeptide Y (MBS2700377), NT-proBNP (MBS2501591), caspase-1 (MBS455768), IL18 (MBS265871) and IL-1β (MBS355354) were measured using commercial ELISA kits (MyBioSource, San Diego, CA, USA) according to the manufacturer’s instructions. After treatment, conditioned medium from H9c2 cells and primary NRCMs was collected for the determination of cytokines IL18 (DY521-05, R&D Systems, Minneapolis, MN, USA), IL-1β (RLB00, R&D Systems, Minneapolis, MN, USA), IL-6 (ab234570, Abcam, Cambridge, United Kingdom), IL-10 (ab214566, Abcam, Cambridge, United Kingdom), and TNF-α (ab236712, Abcam, Cambridge, United Kingdom) via ELISA kits.

### Pharmacological interventions

P2X7 Antagonist (A438079, 899431–18-6, MedChemExpress, Monmouth Junction, NJ, USA): Mice were implanted with subcutaneous ALZET® osmotic pumps (1002, Alzet, Cupertino, CA, USA) delivering A438079 (5 mg/kg/day) for 2 weeks post HFpEF induction. NLRP3 Inhibitor (MCC950, 210826–40-7, MCE, Monmouth Junction, NJ, USA): Similarly, mice received MCC950 (10 mg/kg/day) via ALZET® osmotic pumps for 2 weeks. Vehicle-treated mice served as controls. After drug administration, cardiac remodeling indices, echocardiographic parameters, exercise capacity, and inflammatory markers were assessed.

### Cell viability assay

The viability of H9c2 cells was assessed using the Cell Counting Kit-8 assay (CA1210, Solarbio, Beijing, China), according to the manufacturer's instructions. Cells were seeded in 96-well plates at a density of 5,000 cells per well. Following treatment, 10 μl of CCK-8 reagent was added to each well and incubated for an additional 2 h, after which the optical density at 450 nm was determined using a microplate spectrophotometer.

### Immunohistochemical and immunofluorescent staining

Renal arteries and mouse Heart tissues were fixed in 4% paraformaldehyde, embedded in paraffin, and sectioned at 4 μm. For immunohistochemical staining, sections were incubated with primary antibodies anti-TH (25859-1-AP, Proteintech, Rosemont, IL, USA, 1:200) for renal arteries and anti-4-Hydroxynonenal (4-HNE, 68538-1-Ig, Proteintech, Rosemont, IL, USA, 1:200) and anti-NLRP3 (30109-1-AP, Proteintech, Rosemont, IL, USA, 1:200) for Heart tissues, followed by the appropriate biotinylated secondary antibodies. In a blinded manner, staining intensity was scored as 0 (negative), 1 (weak), 2 (mild), 3 (moderate), or 4 (strong).

Heart sections were incubated with primary antibodies against 8-hydroxy-2′-deoxyguanosine (8-OHdG, HY-P81140, MedChemExpress, Monmouth Junction, NJ, USA,1:100), γ-H2AX (AF5836, Beyotime Biotechnology, Shanghai, China, 1:100), p21 (10355-1-AP, Proteintech, Rosemont, IL, USA, 1:100), and 4-hydroxynonenal (68538-1-Ig, Proteintech, Rosemont, IL, USA, 1:200), followed by appropriate Alexa Fluor–conjugated secondary antibodies; nuclei were counterstained with DAPI. Fluorescent images were acquired on a Zeiss AX10 Imager under identical settings. In a blinded manner, quantification included mean fluorescence intensity (MFI) of 4-HNE and 8-OHdG, the percentage of p21-positive nuclei (p21⁺/DAPI), and the mean number of γ-H2AX foci per nucleus (≥ 5 fields/heart; ≥ 100 nuclei for p21 and γ-H2AX).

H9c2 cardiomyocytes on coverslips were fixed in 4% paraformaldehyde, permeabilized with 0.5% Triton X-100, blocked with 5% BSA, and incubated with anti-NLRP3 (30,109–1-AP, Proteintech; 1:200), followed by an FITC-conjugated secondary antibody and DAPI. Images were captured on a Zeiss AX10 microscope, and MFI was qualitatively evaluated to verify expression.

### Measurement of cellular and mitochondrial ROS production

The intracellular ROS production was measured using the dichlorofluorescin diacetate (DCFH-DA, ID3130, Solarbio, Beijing, China) method as described [56]. Briefly, after treatment, cells in 35 mm confocal culture dishes were incubated with DCFDA for 30 min at 37 °C, followed by immediate observation under a High Intelligent and Sensitve SIM super-resolution microscope (Guangzhou CSR Biotech Co. Ltd, Guangzhou, China) at the excitation/emission wavelength of 480/530 nm. Images were acquired using a 100 × /1.5 NA oil immersion objective (Olympus Corporation, Tokyo, Japan). To specifically assess mitochondrial ROS (mtROS), cells were incubated with MitoSOX™ Red (M36008, Thermo Fisher Scientific, Waltham, MA, USA) at a final concentration of 5 μM for 15 min at 37 °C. After gentle washing with PBS to remove excess dye, mitochondrial fluorescence was detected using the same High Intelligent and Sensitve SIM super-resolution microscope with excitation at 510 nm and emission at 580 nm. Quantification of MitoSOX intensity was used to reflect mitochondrial superoxide generation. All measurements were performed in at least three independent experiments, and fluorescence intensity was analyzed using ImageJ software (v1.53c).

### Mitochondrial morphology assessment using mitotracker staining

Mitochondrial morphology was visualized using MitoTracker™ Red CMXRos (Thermo Fisher Scientific, USA) according to the manufacturer's protocol. Briefly, cells cultured on 35 mm confocal dishes were incubated with MitoTracker Red (200 nM, M46752, Thermo Fisher Scientific, Waltham, MA, USA) in serum-free medium for 30 min at 37 °C, protected from light. Following incubation, cells were gently washed with pre-warmed PBS to remove excess dye, and then immediately imaged using a High Intelligent and Sensitve SIM super-resolution microscope (Guangzhou CSR Biotech Co. Ltd, Guangzhou, China).

### Statistical analysis

Results are presented as mean ± standard deviation (SD). Differences between two groups were analyzed using a two-tailed unpaired Student’s t-test, while one-way or two-way analysis of variance (ANOVA) complemented by Tukey’s post-hoc test was employed for multiple comparisons in experiments with three or more groups. For semiquantitative immunohistochemistry scoring, comparisons across the four groups were performed using the Kruskal–Wallis test followed by Dunn’s post-hoc test for all pairwise comparisons. Statistical significance was established at *P* < 0.05. All experiments included a minimum of three biological replicates. Sample sizes were not predetermined statistically but were estimated based on prior experience, experimental methodology, availability, and the feasibility of achieving statistically significant outcomes. Experimental animals were randomly allocated to each experimental or control group. During the experiments and outcome assessments, investigators were blinded to the group assignments of individual animals.

## Results

### Multi-hit risk factors induce an HFpEF phenotype in mice

**Fig. 1:**
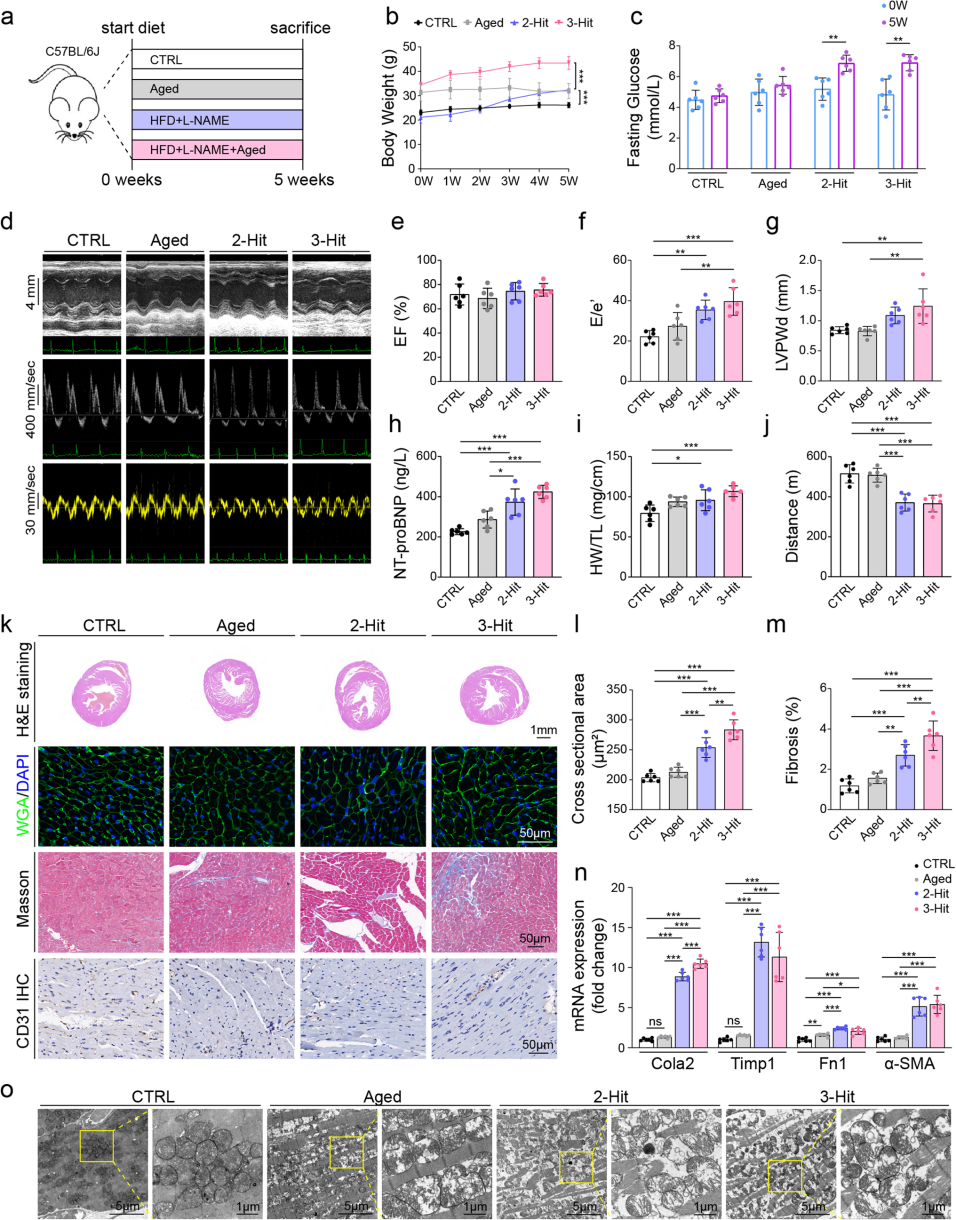
A HFpEF model generated by a muti-Hit strategy. **a** Schematic of animal grouping and modeling over 5 weeks: CTRL, Aged, 2-Hit (HFD + L-NAME), and 3-Hit (HFD + L-NAME + Aged). **b** Body weight changes. **c** Fasting blood glucose at baseline (0 week) and 5 weeks. **d** Representative echocardiographic images (M-mode, pulsed-wave Doppler, and tissue Doppler) from each group. **e** Left ventricular ejection fraction. **f** Ratio of early transmitral flow velocity to early diastolic mitral annular velocity (E/e′). **g** Left ventricular posterior wall thickness at end diastole (LVPWd). **h** Serum NT-proBNP. **i** Heart weight normalized to tibia length (HW/TL). **j** Treadmill running distance measuring exercise capacity. **k** Representative histological images: H&E staining, Wheat Germ Agglutinin (WGA) staining, Masson’s trichrome staining and CD31 immunohistochemistry. **l** Cardiomyocyte cross-sectional area quantified from WGA staining. **m** Quantification of myocardial fibrosis (%) by Masson’s trichrome staining. **n** mRNA expression of Col1a2, Timp1, Fn1, and α-SMA in heart tissue. **o** Transmission electron microscopy of myocardial ultrastructure. One-way ANOVA with Tukey’s post hoc was performed for statistical analysis (**b, e**–**j,** and **l**–**n**). Two-tailed unpaired Student’s *t*-test was performed for statistical analysis (**c**). Data is presented as mean ± SD. n = 6 mice/group. *P < 0.05, **P < 0.01, ***P < 0.001

We established a 3-hit model that combines aging, hypertension and metabolic stress to recapitulate key clinical features of HFpEF (Fig. 1a). The following groups served as controls: CTRL (6-month-old mice, normal diet), Aged (18-month-old mice, normal diet), and 2-hit (6-month-old mice, HFD + L-NAME). After five weeks, both the 3-hit and 2-hit groups exhibited significant increases in body weight (Fig. 1b), fasting blood glucose (Fig. 1c), and blood pressure, including systolic (Fig. S1a) and diastolic pressures (Fig. S1b).

Echocardiography provided representative structural and functional images using B-mode, M-mode and Doppler (Fig. 1d, Fig. S1c). Systolic function was preserved in all groups, with LVEF and fractional shortening (FS) unchanged (Fig. 1e, Fig. S1d). However, the 3-hit and 2-hit groups developed clear signs of diastolic dysfunction, including significantly increased E/e' ratios (Fig. 1f) and mildly elevated E/A ratios (Fig. S1e). Both the 2-hit and 3-hit groups showed greater left ventricular posterior wall thickness during systole and diastole (Fig. S1f, Fig. 1g). Isovolumic relaxation time (IVRT) and the Tei index were higher in both groups as well (Fig. S1h, i). Left ventricular (LV) mass increased in both groups and was higher in the 3-hit group than in the 2-hit group (Fig. S1g).

Circulating NT-proBNP levels, heart weight to tibia length (HW/TL) and lung weight to tibia length (LW/TL) were significantly elevated in both the 3-hit and 2-hit groups, indicative of cardiac remodeling and pulmonary congestion, (Fig. 1h, i and Fig. S1j). Reduced treadmill exercise capacity further confirmed impaired cardiovascular function in both groups (Fig. 1g).

Compared with the 2-hit group, the 3-hit group exhibited more pronounced cardiomyocyte hypertrophy, with larger cross-sectional area (Fig. 1k, l), and showed greater myocardial fibrosis on Masson’s trichrome staining (Fig. 1k, m). Transmission electron microscopy revealed swollen and fragmented mitochondria with disrupted cristae, most prominent in the 3-hit myocardium (Fig. 1o). Capillary density was lower in both the 2-hit and 3-hit groups (Fig. 1k, Fig. S1k). Pro-fibrotic transcripts, including Col1a2, Timp1, Fn1 and α-SMA, were upregulated in both groups, with a larger increase in Col1a2 in the 3-hit group (Fig. 1n). Compared with the 2-hit model, the 3-hit Hearts showed stronger staining for 4-HNE and 8-OHdG, more γH2AX positivity and more p21-positive nuclei, indicating greater oxidative injury, DNA damage and cellular senescence (Fig. S1n–q). Quantification of whole-section H&E images showed a thicker right ventricular (RV) free wall in the 3-hit group than in the 2-hit group (Fig. 1k, Fig. S1l).

Collectively, the 3-hit model reproduces the HFpEF phenotype, including diastolic dysfunction, fibrosis, microvascular rarefaction, and increased LV mass with a thicker RV free wall, with higher oxidative-stress and senescence signatures than 2-hit. Neither aging alone nor 2-hit alone recapitulated the full phenotype.

### Integrated transcriptomic and proteomic profiling reveals immune and metabolic dysregulation in HFpEF hearts

**Fig. 2:**
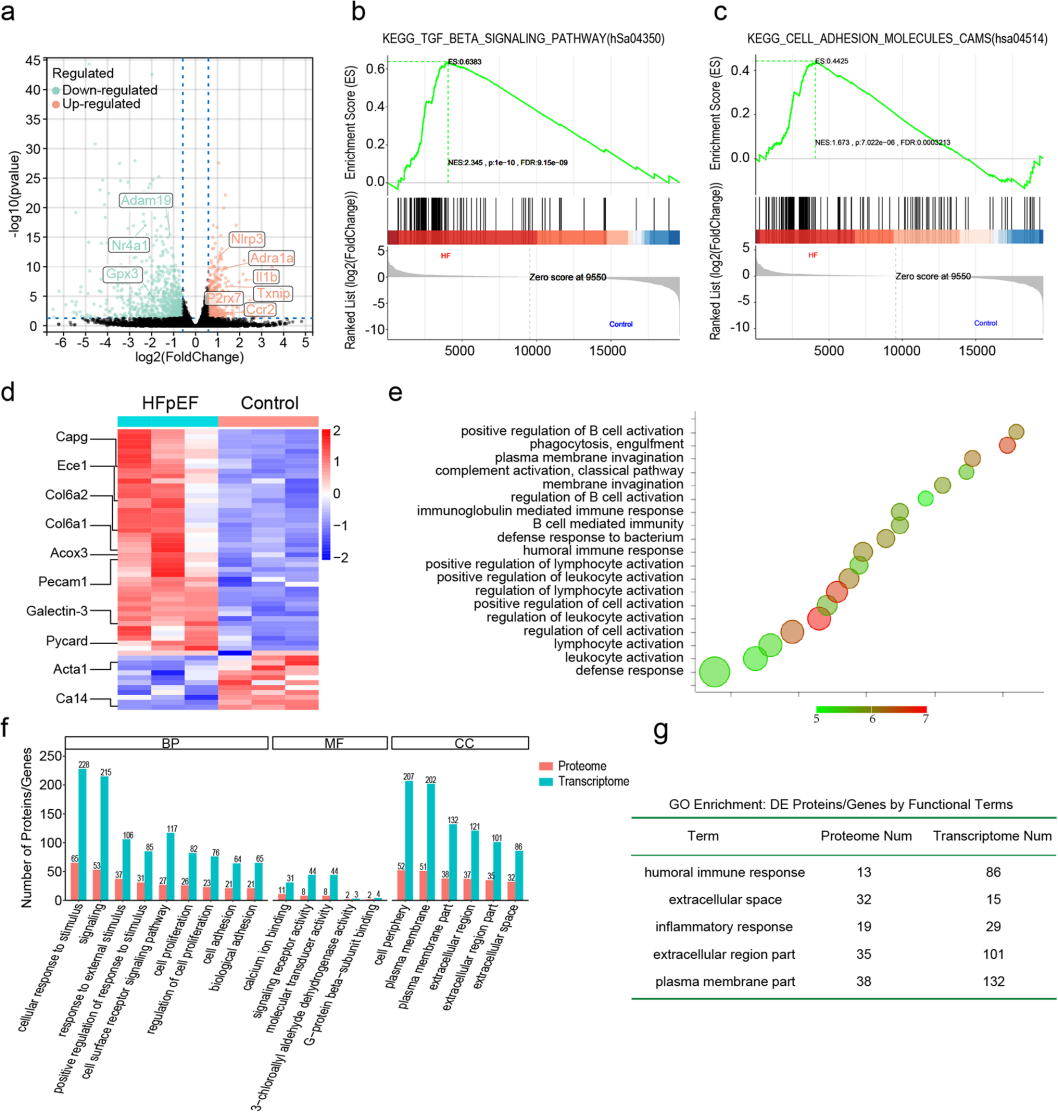
Integrated cardiac transcriptome–proteome analysis identifies inflammation-related pathways in HFpEF. **a** Volcano plot of differentially expressed genes (3-Hit HFpEF vs. Control). **b, c** Gene set enrichment analysis (GSEA) showing significant enrichment of TGF-β signaling (hsa04350) and cell-adhesion molecules (CAMs, hsa04514); green curve, running enrichment score (ES). Normalized enrichment score (NES), nominal *P*, and FDR q are indicated in each panel. **d** Heatmap of selected differentially expressed proteins in HFpEF vs. Control. The color scale (blue to red) indicates lower to higher expression levels. **e** Bubble plot of GO enrichment from the proteomic dataset. The size or color of each bubble generally reflects the enrichment factor or significance level. **f** Top 20 enriched GO terms (biological process, molecular function, cellular component) aggregated across differentially expressed genes and proteins; bars compare the number mapped in the transcriptome (blue) and proteome (orange). **g** Integrated GO terms significantly enriched in both layers; table lists the top five shared categories with counts of differentially expressed proteins and genes. n = 3 mice/group.Significance for omics analyses was assessed using FDR-adjusted *P* values (Benjamini–Hochberg)

To explore the molecular alterations underlying HFpEF pathogenesis, integrated transcriptomic and proteomic analyses were performed on left ventricular tissues from 3-hit HFpEF and control mice. RNA-seq identified broad expression changes. Inflammatory transcripts such as Nlrp3, Adra1e and Il1b increased, whereas redox and metabolic regulators such as Gpx3 and Nr4a1 decreased (Fig. 2a). GSEA showed enrichment of TGF-β signaling and cell adhesion molecules, consistent with fibrotic and inflammatory activation (Fig. 2b, c). Proteomics showed parallel shifts, with collagen subunits Col6a1 and Col6a2, inflammation-related Galectin-3 and Pycard, and cytoskeletal proteins Acta1 and Capg altered in abundance (Fig. 2d). GO analysis of the proteome underscored immune activation, with enrichment of humoral immune response, complement activation and B-cell-mediated immunity (Fig. 2e).

Cross-omics comparative analysis further highlighted concordant changes between transcriptomic and proteomic datasets. Among the top 20 significantly enriched GO terms, immune response, extracellular matrix remodeling, and inflammatory pathways dominated (Fig. 2f). Shared enrichment across both omics included humoral immune response, extracellular space or region, inflammatory response and plasma membrane components (Fig. 2g), indicating a coherent signature of immune and extracellular matrix dysregulation.

Unsupervised clustering of RNA-seq data clearly separated 3-hit HFpEF hearts from non-failing controls, indicating broad transcriptional reprogramming (Fig. S2a). The top transcriptomic GO terms included extracellular matrix organization, response to endogenous stimulus and receptor signaling, consistent with inflammation and fibrosis (Fig. S2b). Additionally, KEGG pathway analysis by GSEA highlighted the leukocyte transendothelial migration pathway, suggesting enhanced immune cell infiltration and endothelial activation in HFpEF myocardium (Fig. S2c). Cross-omics heatmaps and enrichment-correlation plots showed concordant proteomic and transcriptomic changes, with overlapping immune and extracellular matrix pathways (Fig. S2d, e).

Together, multi-omics data identify immune activation and extracellular matrix remodeling as central features of HFpEF, accompanied by metabolic impairment at both gene and protein levels.

### Renal denervation attenuates sympathetic activation and ameliorates HFpEF phenotypes in mice

**Fig. 3:**
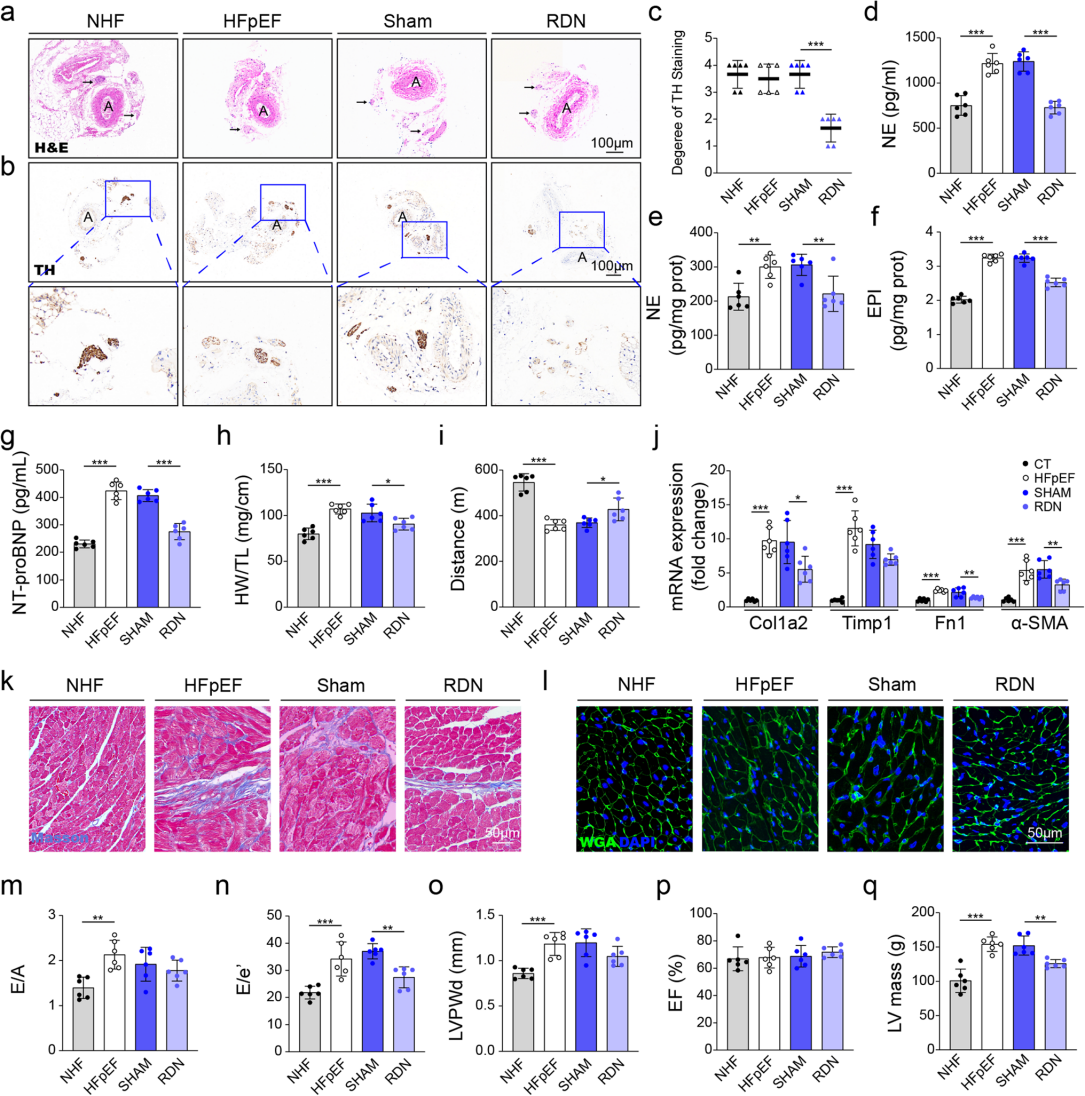
RDN suppresses sympathetic activity and mitigates HFpEF phenotypes. **a** Representative H&E-stained renal artery sections. Arrows indicate nerves; “A” denotes the renal artery lumen. **b** Representative tyrosine hydroxylase (TH)-immunostained renal artery sections. “A” denotes the renal artery lumen. **c** Semi-quantitative scoring of TH staining intensity: 0 (negative), 1 (weak), 2 (mild), 3 (moderate), or 4 (strong). **d**–**f** Norepinephrine (NE) levels in serum (**d**) and heart lysates (**e**), and epinephrine (EPI) levels in heart lysates (**f**). **g** Serum NT-proBNP. **h** Heart weight normalized to tibia length (HW/TL). **i** Exercise capacity measured by treadmill running distance. **j** mRNA expression of Col1a2, Timp1, Fn1, and α-SMA in cardiac tissue. **k** Masson’s trichrome staining showing myocardial fibrosis. **l** WGA staining highlighting cardiomyocyte membranes for cross-sectional area measurements. **m** Ratio of early to late diastolic velocities (E/A). **n** Ratio of early transmitral flow velocity to early diastolic mitral annular velocity (E/e′). **o** Left ventricular posterior wall end diastolic diameter (LVPWd). **p** Left ventricular ejection fraction (EF). **q** Left ventricular mass. Kruskal–Wallis with Dunn’s post hoc was performed for statistical analysis (**c**). One-way ANOVA with Tukey’s post hoc was performed for statistical analysis (**d**–**j,** and **m**–**q**). n = 6 mice/group. Data is presented as mean ± SD. *P < 0.05, **P < 0.01, ***P < 0.001

To test the role of sympathetic hyperactivity in HFpEF, we used naturally aged 18-month-old C57BL/6 J mice as the aging component of the 3-hit model. At week 0, 18-month-old C57BL/6 J mice underwent bilateral RDN or sham. From week 1 to week 6, mice received HFD plus L-NAME for five weeks, and euthanasia was performed at week 6 (Fig. S3a).Renal arteries from denervated mice showed nerve degeneration and disrupted integrity compared with intact nerves in sham controls (Fig. 3a). Immunostaining for TH, a sympathetic nerve marker, confirmed significantly reduced TH-positive nerve fibers following RDN (Fig. 3b, c). Circulating norepinephrine, epinephrine and neuropeptide Y, as well as myocardial norepinephrine and neuropeptide Y, were lower after RDN than in sham-operated HFpEF mice (Fig. 3d-f and Fig. S3b-d). Tail-cuff measurements showed a statistically significant reduction in systolic and diastolic blood pressure at the study endpoint in the RDN group compared with sham (Table S3). Serum creatinine was elevated in HFpEF and was unchanged by RDN (Fig. S3e). RDN notably reduced serum NT-proBNP levels, suggesting alleviation of cardiac stress (Fig. 3g). Heart weight to tibia length and lung weight to tibia length decreased after RDN (Fig. 3h, Fig. S3k). Treadmill endurance improved with RDN (Fig. 3i).

Systolic function was preserved in all groups, with LVEF and FS unchanged (Fig. 3p and Fig. S3f). However, RDN significantly improved diastolic function, evidenced by decreased E/e' ratio, whereas no significant difference in the E/A ratio was observed between the RDN-treated and Sham-treated HFpEF groups (Fig. 3m, n). RDN reduced posterior wall thickness at systole, with no difference at diastole (Fig. 3o, Fig. S3g). LV mass was lower after RDN (Fig. 3q).

Histological examination further supported these functional improvements. WGA staining showed smaller cardiomyocyte cross-sectional area after RDN (Fig. 3l, Fig. S3h). Consistently, Masson’s trichrome staining demonstrated a significant reduction in myocardial fibrosis after RDN, with lower expression of fibrosis-related genes including Col1a2, Timp1, Fn1 and α-SMA (Fig. 3j, k; Fig. S3i). Additionally, cardiac microvascular integrity was improved, as indicated by higher myocardial capillary density in RDN-treated mice compared to Sham-operated HFpEF mice (Fig. S3j).

Together, these data show that sympathetic hyperactivity is associated with cardiac remodeling, diastolic dysfunction and exercise intolerance in HFpEF. Renal denervation lowered sympathetic activity and was accompanied by structural and functional improvements.

### Renal denervation reduces oxidative stress, inflammation and cardiomyocyte injury in HFpEF mice

**Fig. 4:**
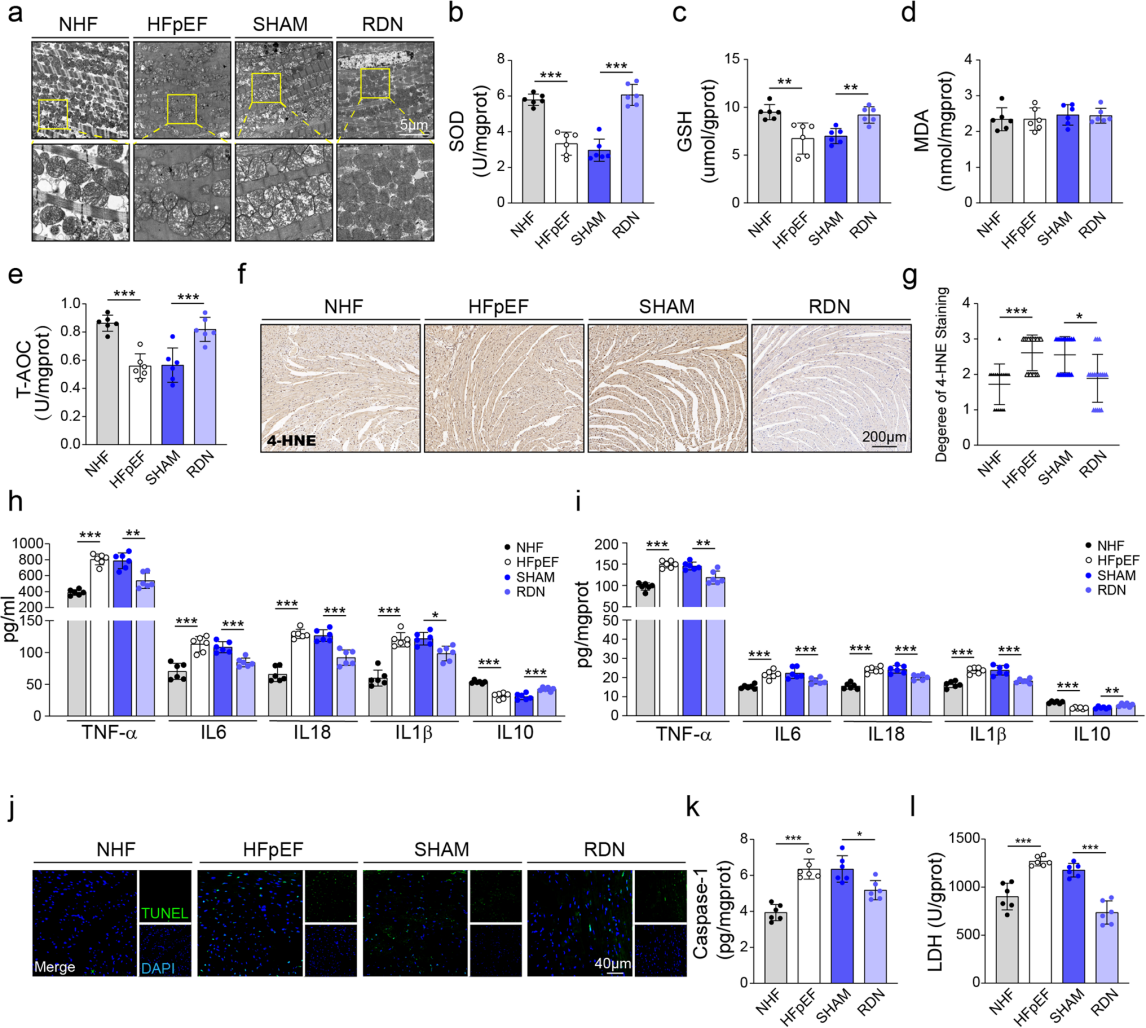
Renal denervation reduces oxidative stress levels and inflammatory responses in HFpEF mice. **a** Representative transmission electron microscopy images of cardiomyocytes. Boxes highlight areas of mitochondrial changes. **b**–**e** Superoxide dismutase (SOD), glutathione (GSH), malondialdehyde (MDA), and total antioxidant capacity (T-AOC) in heart lysates. **f** Immunohistochemical staining of 4-hydroxynonenal (4-HNE) in myocardial sections. **g** Semi-quantitative scoring of 4-HNE staining: 0 (negative), 1 (weak), 2 (mild), 3 (moderate), 4 (strong). **h, i** Pro-inflammatory cytokines (TNF-α, IL-6, IL-18, IL-1β) and the anti-inflammatory cytokine IL-10 in serum (**h**) and heart lysates (**i**). **j** Representative TUNEL staining images of myocardial sections. **k** Caspase-1 levels in heart lysates. **l** Lactate dehydrogenase (LDH) activity in heart lysates. One-way ANOVA with Tukey’s post hoc was performed for statistical analysis. n = 6 mice/group. Data is presented as mean ± SD. *P < 0.05; **P < 0.01; ***P < 0.001

To assess oxidative stress, inflammation and cell injury after RDN, we examined mitochondrial ultrastructure, oxidative and antioxidant markers, inflammatory cytokines and apoptosis. Transmission electron microscopy showed swollen and fragmented mitochondria with disrupted cristae in HFpEF hearts, which were attenuated after RDN (Fig. 4a). Biochemical analyses demonstrated decreased levels of the antioxidants SOD, GSH, and T-AOC in myocardial tissues from HFpEF mice, while RDN treatment markedly restored their levels (Fig. 4b-e). MDA levels, however, showed no significant differences among groups. Immunohistochemistry for 4-HNE, a lipid peroxidation marker, showed higher staining in HFpEF that decreased after RDN (Fig. 4f, g). Similarly, systemic oxidative stress levels evaluated in serum showed a parallel increase in GSH, SOD, and T-AOC after RDN, whereas serum MDA levels remained unchanged (Fig. S4a-d).

Pro-inflammatory cytokines including TNF-α, IL-6, IL-18 and IL-1β were higher, and the anti-inflammatory cytokine IL-10 was lower, in HFpEF than in NHF controls, in both serum and myocardium. RDN reduced pro-inflammatory cytokines and increased IL-10 (Fig. 4h, i). Additionally, myocardial injury was evaluated by apoptosis and cell-damage markers. HFpEF mice exhibited elevated myocardial caspase-1 activity and LDH release compared to NHF controls, both of which were reduced by RDN treatment (Fig. 4k, l). Consistent findings were observed in serum LDH activity and caspase-1 levels (Fig. S4e, f). Furthermore, TUNEL staining showed increased cardiomyocyte apoptosis in HFpEF mice, significantly reduced by RDN intervention (Fig. 4j and Fig. S4g). Collectively, these data demonstrate that RDN effectively alleviates mitochondrial damage, oxidative stress, inflammatory responses, and cardiomyocyte injury, highlighting its protective role in mitigating HFpEF-associated myocardial pathology.

### Renal denervation attenuates ATP-P2X7 axis-mediated NLRP3 inflammasome activation in HFpEF mice

**Fig. 5:**
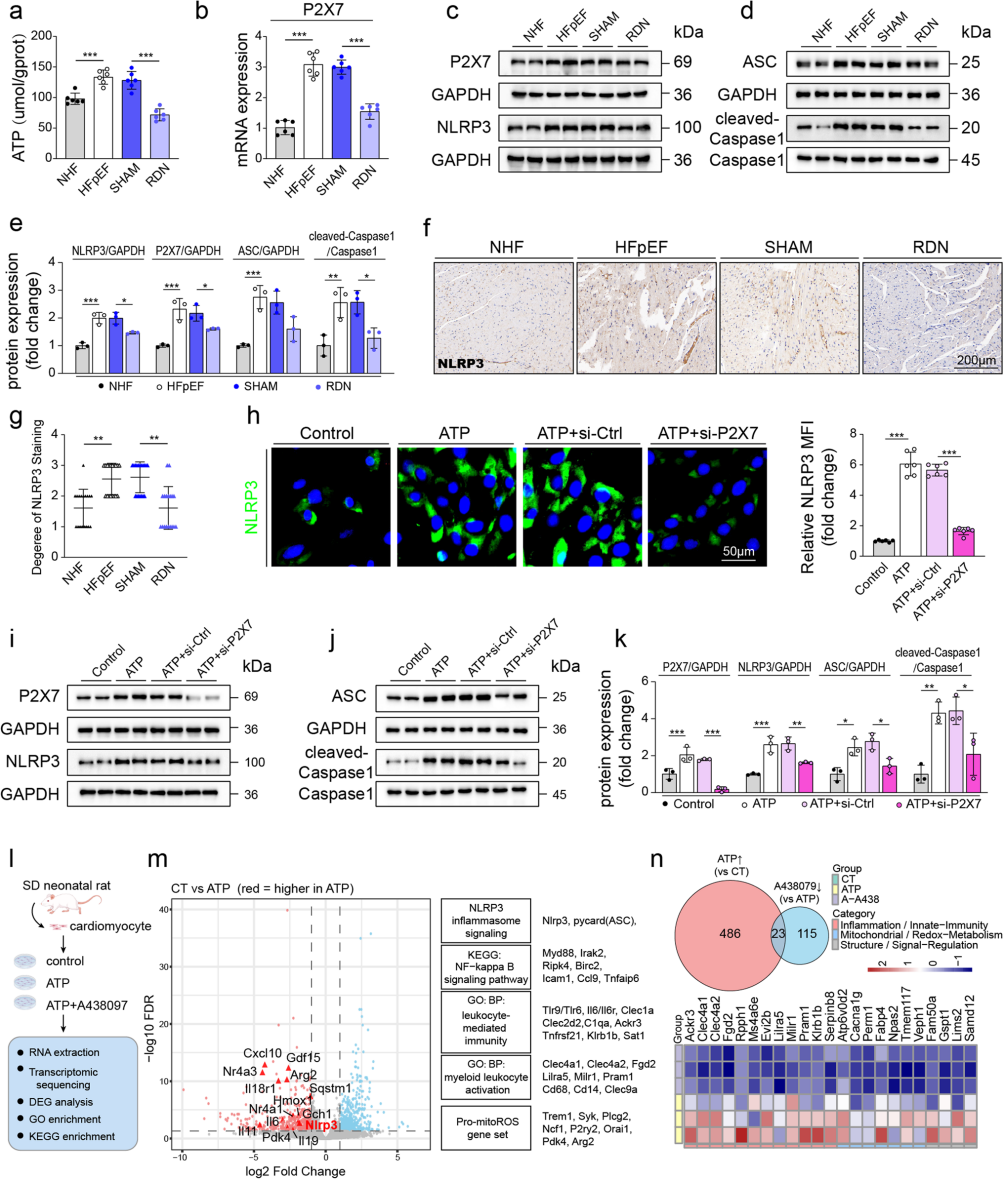
Renal denervation reduces NLRP3 inflammasome activation induced by the ATP-P2X7 axis. **a** ATP levels in heart lysates (n = 6). **b** P2X7 mRNA expression in heart tissue, determined by qRT-PCR (n = 6). **c**, **d** Western blot analysis of P2X7, NLRP3 (**c**), ASC, and cleaved-caspase-1 (**d**) in mouse hearts. **e** Quantification of protein expression levels from (**c**, **d**) (n = 3). **f** Immunohistochemical staining of NLRP3 in myocardial sections. **g** Semi-quantitative scoring of NLRP3 immunoreactivity: 0 (negative), 1 (weak), 2 (mild), 3 (moderate), 4 (strong) (n = 6). **h** NLRP3 immunofluorescence in H9c2 cardiomyocytes transfected with control siRNA (si-Ctrl) or P2X7 siRNA (si-P2X7) and stimulated with ATP (5 mM). Right panel: relative mean fluorescence intensity (MFI) quantification (n = 6). **i**, **j** Western blot of P2X7, NLRP3 (**i**), ASC, and cleaved-caspase-1 (**j**) in H9c2 cells under the same treatments. **k** Quantification of the protein levels shown in (**i**, **j**) (n = 3). **l** Schematic of RNA-seq workflow in neonatal rat ventricular myocytes (NRVMs): Control, ATP, and ATP plus the P2X7 antagonist A438079. **m** Volcano plot of differentially expressed genes for ATP vs. Control; selected genes related to NLRP3 inflammasome/NF-κB/leukocyte activation are highlighted (FDR-adjusted *P* shown on the y-axis). **n** Integrated transcriptomics: Venn diagram showing ATP-induced DEGs reversed by A438079, with a heat map of representative shared genes across groups. One-way ANOVA with Tukey’s post hoc test for (**a, b, e, h** and **k**); Kruskal–Wallis with Dunn’s post hoc for the ordinal IHC score (**g**). Omics significance was assessed by FDR-adjusted *P* values (Benjamini-Hochberg). Data are mean ± SD unless noted. *P < 0.05, **P < 0.01, ***P < 0.001

Given the role of inflammation in HFpEF progression, we further explored whether RDN attenuated cardiac inflammation via the ATP-P2X7-NLRP3 inflammasome pathway. ATP, an essential activator of the P2X7 receptor, was significantly elevated in both myocardial tissues and serum of HFpEF mice compared to NHF mice, whereas RDN effectively reduced ATP levels in cardiac tissue (Fig. 5a) but not significantly in serum (Fig. S4h). Consistently, cardiac mRNA expression of the P2X7 receptor was markedly increased in HFpEF hearts, and significantly suppressed by RDN (Fig. 5b). In myocardium, protein levels of P2X7, NLRP3, ASC and cleaved-caspase-1 were higher in HFpEF and were lower after RDN (Fig. 5c–e). NLRP3 immunohistochemistry showed stronger staining in HFpEF that was lower after RDN (Fig. 5f, g).

To validate the ATP-P2X7-NLRP3 axis mechanistically, we performed cellular experiments using H9c2 cardiomyocytes. Immunofluorescence showed significantly increased NLRP3 expression upon ATP stimulation. However, this increase was markedly attenuated by siRNA-mediated knockdown of P2X7 (Fig. 5h). Supporting these findings, Western blot analysis indicated that ATP treatment upregulated the expression of P2X7, NLRP3, ASC, and cleaved-caspase-1 in H9c2 cells, while silencing P2X7 significantly reduced their expression levels (Fig. 5i–k).

In neonatal rat ventricular myocytes, RNA sequencing across Control, ATP and ATP plus the P2X7 antagonist A438079 is shown in the workflow (Fig. 5l). Compared with Control, ATP induced a broad transcriptional program enriched for the NLRP3 inflammasome and NF-kappa B signaling, with higher transcripts such as Nlrp3 and Pycard, together with immune modules related to leukocyte mediated immunity and myeloid activation (Fig. 5m). A mitochondrial reactive oxygen species related gene set was also upregulated, with representative genes including Ncf1, P2ry2, Orai1, Pdk4 and Arg2 (Fig. 5m). A438079 partially reversed these ATP driven changes, as reflected by the overlap of differentially expressed genes and the shared gene heat map (Fig. 5n). Supplementary analyses were concordant, showing group level expression patterns, a volcano plot for ATP versus A438079 and enrichment of immune and inflammatory pathways for ATP upregulated genes, including cytokine receptor interaction, PI3K-Akt signaling, complement and coagulation cascades, lysosome, phagosome, ferroptosis and NF-kappa B signaling (Fig. S5a-d).

Together, RDN was associated with lower myocardial ATP, reduced P2X7 expression and less NLRP3 inflammasome signaling in vivo, in line with cellular and transcriptomic evidence that ATP activates this pathway and that P2X7 blockade attenuates it.

### Pharmacologic and genetic inhibition of P2X7 mitigates ATP-evoked mitochondrial ROS, oxidative stress and pyroptosis in cardiomyocytes.

**Fig. 6:**
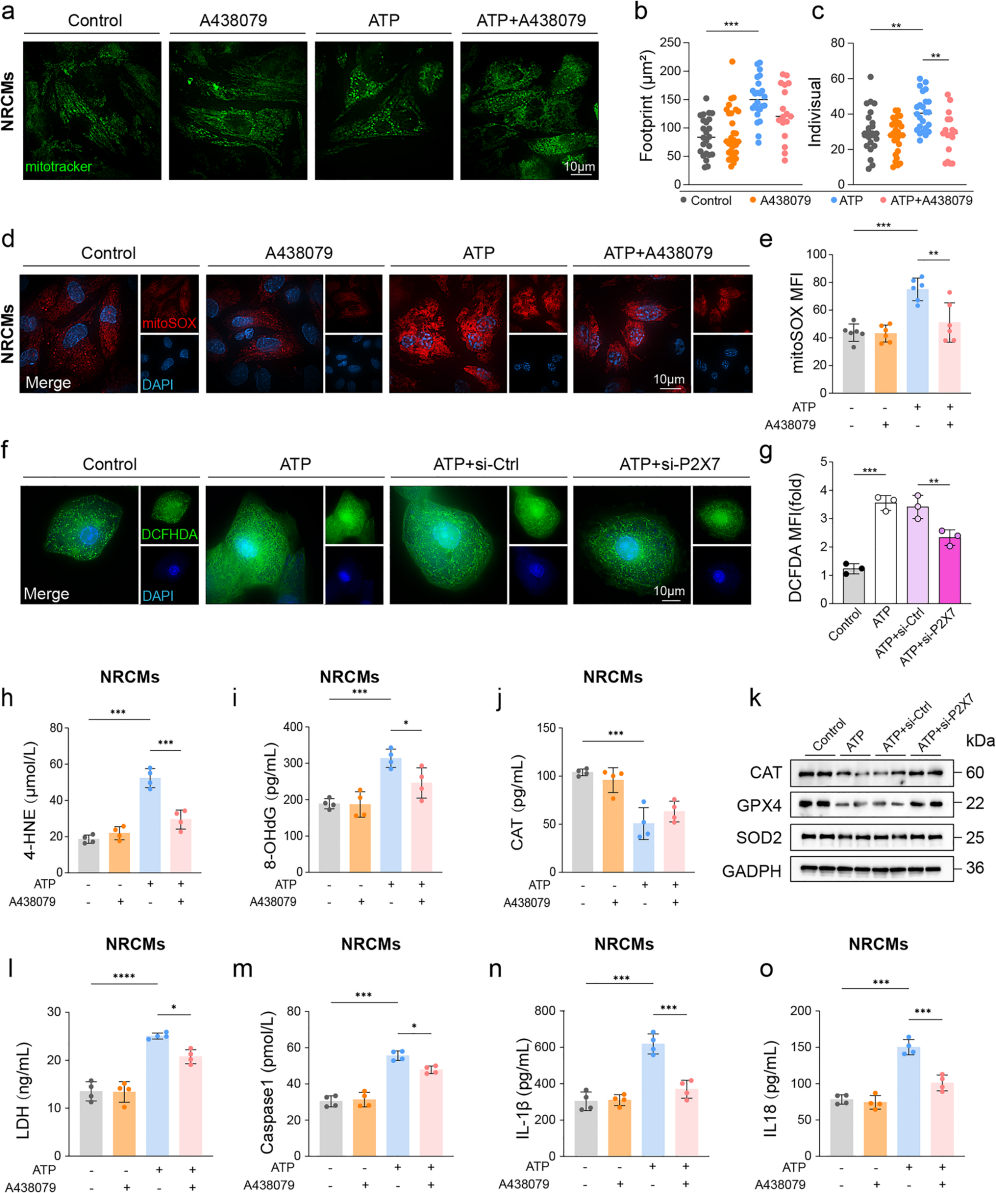
Pharmacologic and genetic inhibition of P2X7 mitigates ATP-evoked mitochondrial ROS, oxidative stress, and pyroptosis in vitro. Neonatal Sprague–Dawley rat cardiomyocytes (NRCMs) were pre-treated with the P2X7 antagonist A-438079 (10 µM, 1 h) and then challenged with ATP (5 mM) for 24 h; vehicle was 0.1% DMSO. An A-438079-alone group was included. H9c2 cardiomyocytes were transfected with control siRNA or P2rx7 (P2X7) siRNA and stimulated with ATP (5 mM, 24 h). **a** MitoTracker staining showing mitochondrial networks under the indicated NRCM treatments. **b, c** Quantification of mitochondrial network metrics in NRCMs: footprint (µm^2^) (**b**) and individuals (**c**). **d** Representative MitoSOX staining (with DAPI) visualizing mitochondrial superoxide in NRCMs. **e** MitoSOX mean fluorescence intensity (MFI) in NRCMs (n = 6). **f** Representative DCFH-DA fluorescence images of intracellular ROS (green) with DAPI nuclear counterstain (blue) in H9c2 cardiomyocytes. **g** Quantification of DCFH-DA mean fluorescence intensity in H9c2 cardiomyocytes (MFI, fold to Control) (n = 3). **h-j** Oxidative-stress markers in NRCMs: 4-hydroxynonenal (4-HNE) (**h**), 8-OHdG (**i**), catalase (CAT) (**j**) (n = 4). **l** Western blots of key antioxidant enzymes (CAT, GPX4, SOD2) in H9c2 cells under siRNA treatments. **l**–**o** Cytotoxicity/pyroptosis readouts in NRCMs subjected to the pharmacologic protocol: LDH release (**l**), caspase-1 activity (**m**), and IL-1β (**n**) and IL-18 (**o**) in supernatants (n = 4). One-way ANOVA with Tukey’s post hoc was performed for statistical analysis. Data is presented as mean ± SD. *P < 0.05; **P < 0.01; ***P < 0.001

We used NRCMs pretreated with the P2X7 antagonist A438079 and H9c2 cells transfected with P2X7 siRNA or control siRNA, then challenged cells with ATP. In NRCMs, ATP disrupted the mitochondrial network, with reduced footprint and more fragmented individuals, and A438079 improved both metrics (Fig. 6a–c). MitoSOX staining showed higher mitochondrial superoxide after ATP that decreased with A438079 (Fig. 6d, e). In H9c2 cells, P2X7 knockdown similarly preserved mitochondrial networks (Fig. S6c). In H9c2 cells, ATP markedly elevated intracellular reactive oxygen species as detected by increased DCFH-DA fluorescence intensity, while P2X7 knockdown significantly reduced ATP-induced reactive oxygen species generation (Fig. 6f, g). In NRCMs, A438079 reduced ATP-induced increases in 4 HNE and 8 OHdG and increased CAT relative to ATP (Fig. 6f–h). In H9c2 cells, western blot analyses indicated reduced expression of key antioxidant enzymes (CAT, GPX4, SOD2) following ATP stimulation, all of which were partially restored after si-P2X7 treatment in H9c2 cells (Fig. 6k).

To evaluate cellular injury and pyroptosis, LDH and caspase-1 activities were measured. In NRCMs, ATP increased LDH release and caspase 1 activity, and A438079 decreased both (Fig. 6i, m). In H9c2 cells, ATP similarly increased LDH release and caspase 1 activity, and P2X7 knockdown attenuated both increases (Fig. S6e, f). In NRCMs, ATP treatment increased secretion of pyroptosis-related cytokines IL-18 and IL-1β, which were notably reduced by A438079 (Fig. 6n, o). In parallel, ATP raised IL-1β and IL-18, and P2X7 knockdown reduced each in H9c2 cells (Fig. S6g, h). Complementary analyses confirmed that ATP exposure significantly compromised cardiomyocyte viability, as evidenced by the CCK-8 assay, whereas P2X7 knockdown restored cell survival (Fig. S5i). In H9c2 cells, ATP increased the pro-inflammatory cytokines IL-6 and TNF-α and lowered the anti-inflammatory cytokine IL-10, and P2X7 knockdown reversed these changes (Fig. S5l-n).

Overall, ATP was associated with increased oxidative stress, disrupted mitochondrial networks, pyroptosis and elevated cytokine release. Both A438079 and P2X7 knockdown were associated with reductions in these measures.

### Pharmacological inhibition of P2X7 with A438079 improves HFpEF phenotypes in mice

**Fig. 7:**
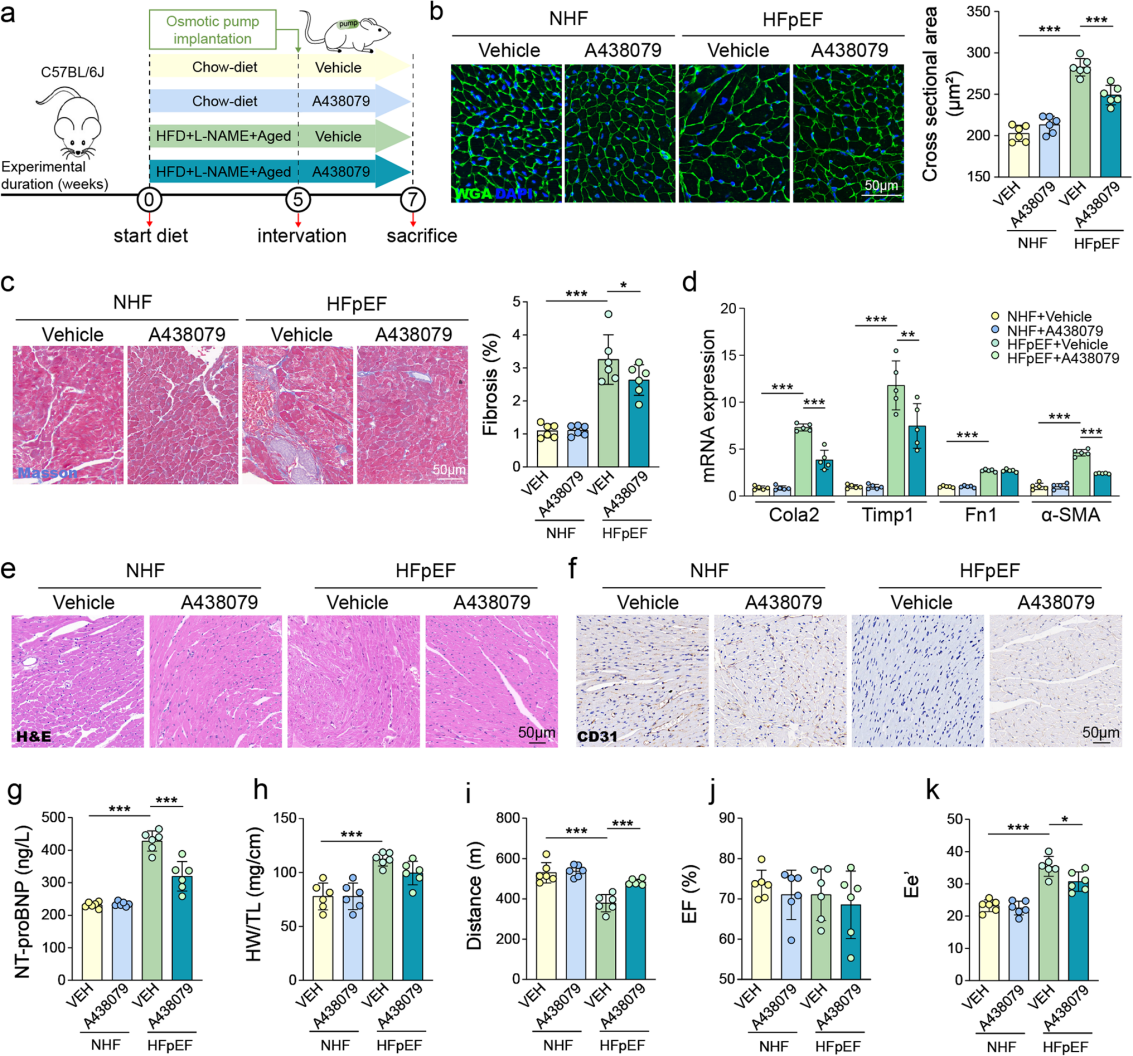
Pharmacological inhibition of P2X7 by A438079 improves HFpEF phenotypes. **a** Schematic of the experimental design. After 3-hit HFpEF induction, mice received A438079 (5 mg/kg, via osmotic pump) or vehicle for two weeks. **b** WGA staining of myocardial sections showing cross-sectional area of cardiomyocytes, quantified on the right. **c** Masson’s trichrome staining to evaluate myocardial fibrosis, with fibrosis percentage shown on the right. **d** mRNA expression of Col1a2, Timp1, Fn1, and α-SMA in hearts. **e** Representative H&E staining of heart sections. **f** Representative images of CD31 immunohistochemical staining in heart sections. **g** Serum NT-proBNP. **h** Heart weight normalized to tibia length (HW/TL). **i** Treadmill running distance for exercise capacity. **j** Left ventricular ejection fraction measured by echocardiography. **k** Ratio of early Doppler transmitral flow velocity (E) to tissue Doppler velocity (e′). One-way ANOVA with Tukey’s post hoc was performed for statistical analysis. n = 6 mice/group. Data is presented as mean ± SD. *P < 0.05, **P < 0.01, ***P < 0.001

To evaluate the therapeutic potential of pharmacological P2X7 inhibition in HFpEF, mice subjected to the 3-hit HFpEF model received the selective P2X7 antagonist A438079 (5 mg/kg/day, via osmotic pumps) for two weeks following disease induction (Fig. 7a). Histological analyses demonstrated significant cardiac remodeling in vehicle-treated HFpEF mice, including cardiomyocyte hypertrophy and fibrosis. WGA staining showed markedly enlarged cardiomyocyte cross-sectional area in HFpEF mice compared to controls, which was significantly reduced by A438079 treatment (Fig. 7b). Masson's trichrome staining indicated prominent myocardial fibrosis in HFpEF hearts, also significantly attenuated by A438079 administration (Fig. 7c). Correspondingly, gene expression levels of fibrotic markers (Col1a2, Timp1, Fn1, α-SMA) were markedly elevated in HFpEF hearts, with significant reduction observed after A438079 treatment (Fig. 7d). Histological examination via H&E staining confirmed improvements in cardiac tissue morphology following P2X7 inhibition (Fig. 7e). Additionally, CD31 immunostaining indicated increased capillary density and improved myocardial vascular integrity after A438079 administration in HFpEF mice (Fig. 7f).

Systemic and functional assessments supported the beneficial effects of P2X7 blockade. Serum NT-proBNP levels were significantly elevated in vehicle-treated HFpEF mice, indicative of cardiac stress, and these levels were substantially reduced by A438079 (Fig. 7g). HW/TL ratios were significantly elevated in HFpEF mice but were not significantly altered by P2X7 inhibition (Fig. 7h). Functional evaluation demonstrated that exercise capacity was significantly impaired in HFpEF mice and notably improved after A438079 treatment (Fig. 7i). Echocardiographic analysis showed preserved LVEF across all groups, consistent with the clinical definition of HFpEF (Fig. 7j). However, diastolic dysfunction was evident in vehicle-treated HFpEF mice, as reflected by significantly increased E/e' ratios, which were notably decreased after A438079 treatment, indicating improved diastolic relaxation (Fig. 7k).

Overall, these findings indicate that pharmacological inhibition of P2X7 receptor signaling by A438079 effectively ameliorates structural, molecular, and functional abnormalities associated with HFpEF in mice.

### NLRP3 inflammasome inhibition by MCC950 ameliorates HFpEF-related cardiac dysfunction

**Fig. 8:**
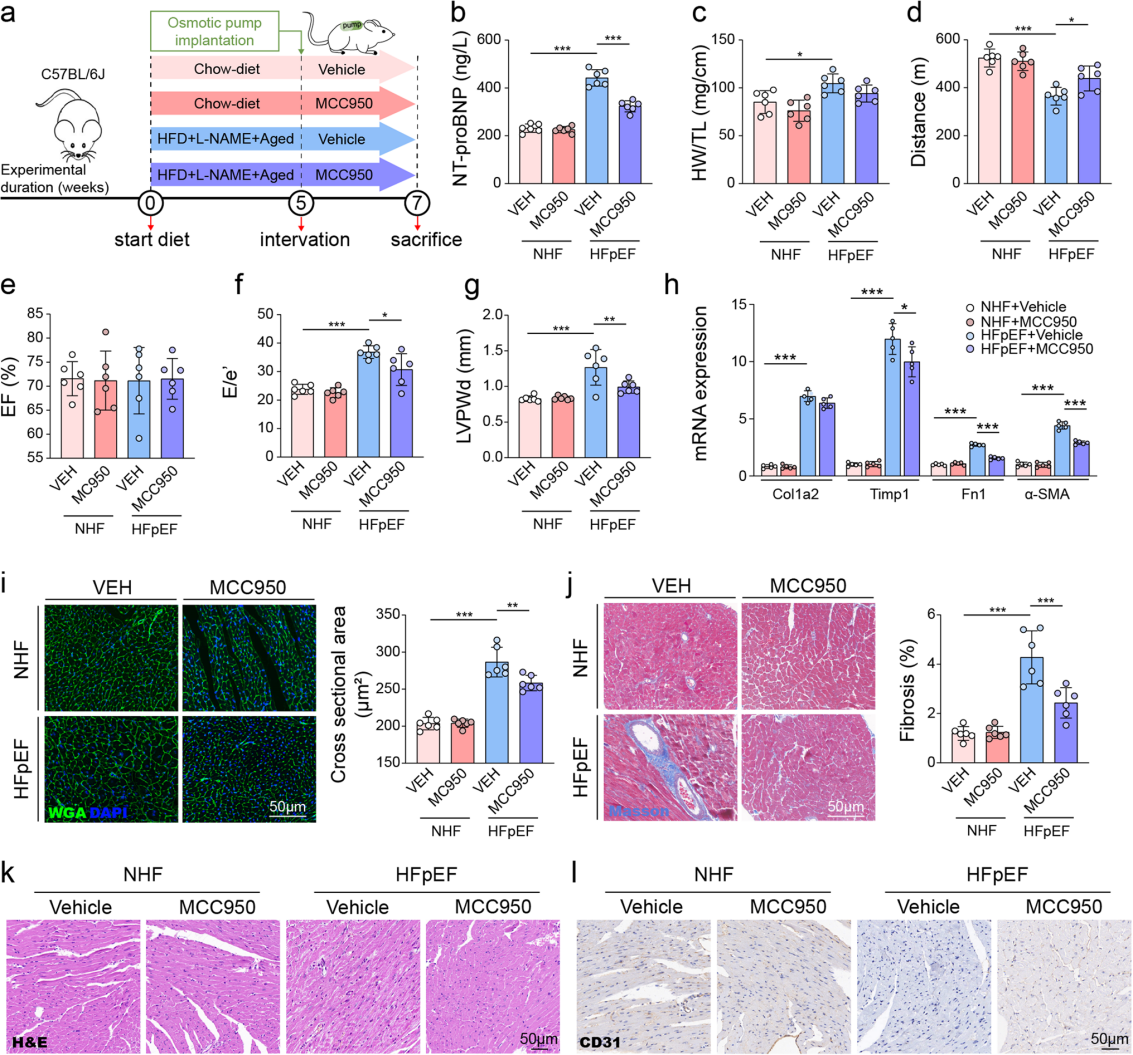
NLRP3 inflammasome inhibition by MCC950 alleviates HFpEF-related cardiac dysfunction. **a** Overview of the experimental protocol. After 3-hit HFpEF induction, mice were treated with MCC950 (10 mg/kg, via osmotic pump) or vehicle for two weeks. **b** Serum NT-proBNP levels. **c** Heart weight normalized to tibia length (HW/TL). **d** Treadmill distance for exercise performance. **e, f** Echocardiographic assessment of left ventricular ejection fraction and E/e′ ratio. **g** Left ventricular posterior wall end-diastolic diameter (LVPWd). **h** mRNA expression of Col1a2, Timp1, Fn1, and α-SMA in hearts. **i** WGA staining showing cardiomyocyte cross-sectional area, with quantification on the right. **j** Masson’s trichrome staining for fibrosis assessment, with the percentage fibrosis on the right. **k** Representative H&E staining of heart sections. **l** CD31 immunostaining for endothelial cells. One-way ANOVA with Tukey’s post hoc was performed for statistical analysis. n = 6 mice/group. Data is presented as mean ± SD. *P < 0.05; **P < 0.01; ***P < 0.001

To investigate whether targeting the NLRP3 inflammasome could mitigate HFpEF phenotypes, HFpEF mice were administered MCC950 (10 mg/kg/day, via osmotic pumps) or vehicle control for two weeks after model induction (Fig. 8a). Serum NT-proBNP, a biomarker of cardiac stress, was significantly elevated in HFpEF mice compared to controls, and MCC950 treatment markedly decreased these levels (Fig. 8b). Additionally, HW/TL was significantly increased in HFpEF mice, but it was not significantly changed by MCC950 treatment (Fig. 8c). Functional assessments revealed impaired exercise capacity in HFpEF mice, as demonstrated by reduced treadmill running distances. MCC950 significantly improved exercise performance in HFpEF animals compared with vehicle-treated counterparts (Fig. 8d).

Echocardiographic evaluations showed preserved LVEF across all groups (Fig. 8e). However, HFpEF mice exhibited significant diastolic dysfunction indicated by elevated E/e' ratios and increased left ventricular posterior wall thickness in diastole. Both of these parameters were significantly improved by MCC950 administration, indicating reduced diastolic impairment and cardiac remodeling (Fig. 8f, g). Quantitative PCR analysis revealed that HFpEF mice exhibited elevated expression of pro-fibrotic genes (Col1a2, Timp1, Fn1, α-SMA). MCC950 treatment significantly lowered Timp1, Fn1 and α-SMA levels, whereas Col1a2 expression was not significantly affected (Fig. 8h).

Histological analysis further confirmed cardiomyocyte hypertrophy and fibrosis in HFpEF hearts. WGA staining showed enlarged cardiomyocyte cross-sectional areas significantly reduced by MCC950 treatment (Fig. 8i). Similarly, Masson’s trichrome staining indicated marked myocardial fibrosis, which was significantly attenuated by MCC950 (Fig. 8j). Histological examination via H&E staining indicated improved myocardial structure following MCC950 administration (Fig. 8k). Additionally, CD31 immunostaining revealed enhanced capillary density and improved microvascular integrity after inflammasome inhibition (Fig. 8l).

Collectively, these data demonstrate that pharmacological inhibition of the NLRP3 inflammasome by MCC950 alleviates myocardial remodeling, attenuates fibrosis and hypertrophy, and significantly improves cardiac function and exercise capacity in mice with HFpEF.

## Discussion

HFpEF is a multifaceted clinical syndrome arising from the interplay of various comorbidities, notably advanced age, metabolic syndrome, and chronic hypertension. These factors collectively produce a systemic condition marked by multi-organ dysfunction involving the heart, lungs, skeletal muscles, and vascular [13]. This complexity has made it challenging to develop animal models that faithfully replicate the human HFpEF phenotype, thereby Limiting mechanistic insights and impeding therapeutic advances. In this study, we expanded the established 2-hit HFpEF model (HFD plus L-NAME) [47] by incorporating biological aging, thereby creating a comprehensive 3-hit model that more closely recapitulates the human HFpEF condition. This approach aligns with the current understanding that HFpEF arises from the synergistic effects of multiple coexisting comorbidities. Compared with the original 2-hit model, our 3-hit model reproduced the key features of HFpEF and additionally showed more severe pathology. This included more extensive interstitial fibrosis, higher levels of oxidative stress and DNA damage markers (4-HNE, 8-OHdG, and γH2AX), an increased number of p21-positive senescent cardiomyocytes, and a thicker RV free wall. These findings indicate that the added aging “hit” amplifies oxidative stress, DNA damage and cellular senescence, fibrosis, and secondary right-ventricular remodeling, thereby providing a clinically meaningful enhancement of the conventional 2-hit model. Ultimately, this underscores the necessity of incorporating multiple risk factors to accurately reflect the complex pathophysiology observed in HFpEF patients.

Studies have demonstrated that SNS activation is closely linked to the pathophysiological processes driving cardiovascular diseases, particularly in elderly individuals and patients with heart failure or metabolic syndrome, conditions typically accompanied by elevated sympathetic tone [42]. SNS activation impacts not only cardiovascular function but may also exacerbate disease progression through modulation of inflammatory and metabolic pathways [12, 28, 32, 45]. Indeed, recent evidence indicates that SNS overactivity, mediated via α_1_-adrenergic receptors, can amplify inflammatory responses, contributing to conditions beyond cardiovascular disease [4]. Moreover, sustained sympathetic hyperactivation plays a crucial role in heart failure progression, which is a multifactorial syndrome marked by pronounced neurohormonal imbalance, including upregulated SNS drive. Chronic SNS stimulation induces detrimental effects such as cardiomyocyte apoptosis, adverse ventricular and vascular remodeling, and heightened arrhythmogenic risk, collectively accelerating the decline of cardiac function [15]. In line with the pathogenic role of SNS overactivity, previous studies showed that early RDN can ameliorate HFpEF in Zucker fatty obese rats [18]. In our study using a clinically relevant 3-hit mouse model of HFpEF, RDN significantly alleviated diastolic dysfunction and cardiac remodeling, evidenced by reduced cardiomyocyte hypertrophy and interstitial fibrosis, improved ventricular compliance, and enhanced exercise tolerance. Most previous studies of RDN have examined its antihypertensive effects in resistant hypertension and its therapeutic benefits in HFrEF [5, 8, 19]. In our experiments, RDN induced a statistically significant but modest reduction in arterial pressure, and both our data and prior reports indicate that this reduction alone cannot account for the full extent of functional and structural improvements. Indeed, several preclinical and clinical studies have shown that RDN attenuates ventricular remodeling, oxidative stress, and inflammation even when the accompanying blood pressure decrease is small [38, 44]. Specifically, in our HFpEF model RDN markedly lowered systemic and myocardial norepinephrine, epinephrine, and neuropeptide Y levels, confirming effective attenuation of sympathetic drive. This neurohormonal shift paralleled significant reductions in myocardial oxidative stress markers, pro-inflammatory cytokines, and interstitial fibrosis, all of which are recognized blood pressure–independent mediators of HFpEF progression.

Although RDN in our study was instituted one week before initiating the HFpEF-inducing hits, this preventive design reflects a plausible clinical situation. Patients qualifying for RDN in trials such as SYMPLICITY HTN-3 and SPYRAL HTN-ON MED [1, 35], that is, individuals with resistant hypertension despite multi-drug therapy, often exhibit heightened sympathetic drive and are at high cardiovascular risk. Although prospective data linking this population to incident HFpEF are limited, hypertension is the most prevalent risk factor for HFpEF, with ~ 75–90% of patients reporting a history of hypertension [14]. Moreover, apparent treatment-resistant hypertension is more common in HFpEF than in HFrEF (~ 17% vs ~ 10%) [29]. Taken together, these observations support the clinical relevance of considering sympathomodulation in the HFpEF context; accordingly, early modulation, for example via RDN, could delay or prevent progression to HFpEF, and our findings provide mechanistic support for this concept. Finally, because HFpEF increasingly appears to involve coronary microvascular disease, it is notable that in our model capillary density (CD31-positive microvessels) was reduced versus controls and partially restored by RDN, consistent with microvascular impairment as a hallmark of HFpEF and suggesting a microcirculatory benefit of sympathomodulation [26].

ATP is an essential signaling molecule closely associated with SNS activation. Released primarily from sympathetic nerve terminals, ATP acts on purinergic P2 receptors to regulate renal medullary blood flow, a process crucial for urine concentration and nutrient delivery in the kidney [21]. Furthermore, co-released ATP and norepinephrine synergistically induce vasoconstriction via P2X1 receptors in mesenteric arteries, an effect further modulated by neuropeptide Y [36]. In the context of heart failure, astrocyte-derived ATP in the rostral ventrolateral medulla enhances sympathetic neuronal excitability, thereby exacerbating sympathetic outflow and disease progression [2]. Additionally, ATP in the central nervous system interacts with AMPA/kainate and P2X receptors to augment sympathetic activity and elevate blood pressure [51]. These findings underscore the multifaceted role of ATP in both peripheral and central regulation of sympathetic function through multiple receptor pathways. Beyond its role as a neurotransmitter, extracellular ATP also critically regulates inflammatory responses in cardiomyocytes via P2X7 receptor signaling. Activation of the P2X7 receptor by ATP triggers potassium efflux and calcium influx, which are key events required for the assembly of the NLRP3 inflammasome and subsequent activation of caspase-1, ultimately leading to pyroptotic cell death [23, 33]. In the present study, we demonstrated that RDN significantly decreased myocardial and systemic ATP levels, inhibited the myocardial expression of P2X7 and NLRP3 proteins, and effectively attenuated inflammasome-dependent pyroptosis. Collectively, our findings suggest that excessive sympathetic stimulation exacerbates myocardial injury through purinergic (ATP-mediated) signaling and inflammasome activation, highlighting this axis as a promising therapeutic target in HFpEF.

To further substantiate the role of the ATP-P2X7-NLRP3 cascade in cardiac dysfunction, we performed pharmacological interventions directed at this pathway. Treatment with the selective P2X7 antagonist A-438079 markedly reduced cardiomyocyte hypertrophy, myocardial fibrosis, and inflammatory markers, and improved diastolic function and exercise capacity. The NLRP3 inflammasome inhibitor MCC950 likewise attenuated myocardial inflammation and structural remodeling and improved cardiac function. These findings are consistent with reports that pharmacological inhibition of the NLRP3 inflammasome mitigates cardiac fibrosis and pathological remodeling [48, 52, 53]. Several observations in this study strengthen a causal inference for ATP-P2X7-NLRP3 signaling in HFpEF. First, ATP elevation and NLRP3 inflammasome activation were present in HFpEF hearts, and both pharmacological inhibition with A-438079 and P2X7 knockdown in cardiomyocytes reduced inflammasome activation and improved injury phenotypes. Second, ATP alone induced mitochondrial oxidative injury, cytokine release, and membrane damage in NRCMs and in H9c2 cells, and these effects were attenuated by selective P2X7 antagonism or genetic suppression of P2X7. Third, in vivo coherence across models was evident, because RDN lowered myocardial ATP and P2X7-NLRP3 activation and improved cardiac remodeling and function. Taken together, these convergent interventions and outcomes support a contributory causal role of the ATP-P2X7-NLRP3 axis in HFpEF rather than a purely correlative association. We acknowledge that other pathways are likely involved, and definitive proof will require targeted in vivo loss-of-function studies, such as cardiomyocyte-specific deletion of P2X7 or NLRP3. In this work, RDN provided broader cardioprotection by modulating sympathetic drive and inflammatory activation at the same time. Combining RDN with targeted inflammasome inhibition may therefore yield additive or synergistic benefit, particularly in patients with persistent inflammation or treatment-resistant HFpEF.

Integrated transcriptomic and proteomic analyses in our study further highlighted inflammation and mitochondrial metabolic dysfunction as pivotal contributors to HFpEF pathogenesis. We observed that the differentially expressed genes and proteins in the HFpEF hearts converged predominantly on inflammatory signaling pathways (e.g., complement activation and cytokine cascades) and extracellular matrix remodeling processes (e.g., heightened collagen deposition and fibrosis-associated proteins), alongside a marked downregulation of mitochondrial oxidative phosphorylation components. These molecular patterns reflect the concept of “metainflammation,” which emphasizes the intricate interplay between metabolic disturbances and chronic inflammatory responses in HFpEF [31]. Mitochondrial oxidative stress directly injures cardiomyocytes and, by disturbing mitochondrial dynamics, amplifies myocardial inflammation and fibrosis [57]. In keeping with contemporary redox perspectives that reactive oxygen species also carry cardioprotective signals and that indiscriminate scavenging has repeatedly failed in clinical translation, upstream modulation of sympathetic/ATP-P2X7 signaling may selectively suppress pathological mitochondrial ROS while preserving physiological redox homeostasis, thereby interrupting the feed-forward loop linking mtROS, inflammation, and fibrosis [25]. Moreover, mitochondrial dysfunction promotes cardiac fibrosis by disrupting intracellular calcium homeostasis and energy metabolism, a process that contributes substantially to diastolic impairment in HFpEF [16, 40]. Accordingly, strategies that combine upstream sympathomodulation with interventions to restore mitochondrial function and correct metabolic and inflammatory derangements represent a promising avenue for HFpEF management.

For translation to clinical practice, our study suggests using RDN or similar sympathomodulatory interventions to combat HFpEF in its early stages. RDN has already shown promise in related cardiovascular conditions, including pulmonary hypertension and right ventricular diastolic dysfunction [11]. These disorders share core features with HFpEF, including sympathetic overactivation and impaired diastolic function, which suggests that the benefits of RDN may extend across cardiovascular syndromes driven by similar mechanisms. Timely RDN and future less-invasive methods of sympathetic inhibition could serve as upstream interventions to slow or prevent the evolution of HFpEF in populations with high sympathetic drive, particularly those with resistant hypertension. Given the heterogeneity of HFpEF and the current lack of consistently effective treatments, translation to practice will require careful patient selection and further study.

Importantly, HFpEF is heterogeneous, and patients with clear evidence of sympathetic overactivation, systemic inflammation, or metabolic disturbances may derive the greatest benefit from therapies that target these neuro-inflammatory axes. Our results indicate that RDN, already an established treatment for resistant hypertension, could be repurposed for HFpEF because it concurrently attenuates sympathetic hyperactivity and inflammasome-driven myocardial inflammation. Biomarkers such as elevated ATP levels or pro-inflammatory cytokines may help to identify patient subgroups most likely to respond to RDN or inflammasome inhibitors. This precision approach could enable more personalized and effective management of this challenging syndrome.

We note several limitations of our study. First, all experiments used male mice. As HFpEF disproportionately affects older women and aging may exacerbate the female phenotype, our findings require validation in aged female groups. Incorporating females will also allow us to delineate potential sex-specific signaling responses. Second, our interventions were of relatively short duration (5 weeks for RDN and 2 weeks for drug treatments), which may not fully capture long-term therapeutic effects or late-stage phenomena like sympathetic nerve reinnervation. Third, despite our model’s fidelity to many HFpEF features, fundamental species differences between mice and humans must be considered when extrapolating these results to patients. Fourth, we did not directly assess coronary circulation or coronary blood flow (e.g., coronary flow reserve, microvascular function, or perfusion), which limits interpretation of myocardial perfusion and its coupling to inflammation in this model. Lastly, the ATP-P2X7-NLRP3 pathway examined here is only one component of the complex network of inflammatory and metabolic factors in HFpEF, and other contributory pathways remain to be investigated.

In summary, this work identifies chronic sympathetic overactivation and inflammasome-mediated myocardial inflammation as key drivers of HFpEF. We showed that renal denervation significantly attenuates the HFpEF phenotype in a murine multi-hit model, largely by suppressing ATP-P2X7-NLRP3 inflammasome signaling and its downstream fibrotic and inflammatory effects. Our 3-hit model of HFpEF, incorporating multiple risk factors, offers a valuable platform for dissecting disease mechanisms and evaluating new treatments. Moving forward, research should explore sex-specific responses, long-term outcomes, and combined therapeutic strategies that target the intersection of neural and inflammatory pathways, with the goal of better managing this complex and increasingly prevalent form of heart failure.

## Supplementary Information

Below is the link to the electronic supplementary material.

Supplementary file1 (DOCX 10060 KB)

## Data Availability

The data supporting the findings of this study are available from the corresponding author upon reasonable request.
