# Supplementary material for: Renal denervation attenuates cardiac dysfunction in HFpEF by inhibiting the ATP-P2X7-NLRP3 inflammasome axis
Source: Basic Res Cardiol. 2025 Sep 16;120(6):1225–47. doi: 10.1007/s00395-025-01138-5 (PMC12680762; doi:10.1007/s00395-025-01138-5)
Supplement: Supplementary file 1 — Supplementary file1 (DOCX 10060 KB) [file 395_2025_1138_MOESM1_ESM.docx]

**BASIC RESEARCH IN CARDIOLOGY**

**Supplementary Materials**

**Renal Denervation Attenuates Cardiac Dysfunction in HFpEF by Inhibiting the ATP-P2X7-NLRP3 Inflammasome Axis**

Zhuqing Li ^1, 2, 3, *^, Xiaoqiang Sun ^1, *^, Yanxin Wang ^3, *^, Feng Zhang ^3^, Li Wang ^1^, Chunbo Ai ^2^, Xu Zhang ^1^, Xuemei Yin ^1^, Chunlei Liu ^4^, Chao Li ^1, #^, Chengzhi Lu ^1, 4, #^

^1^ Department of Cardiology, Tianjin First Central Hospital, Tianjin 300192, China

^2^ Department of Physiology and Biomedical Engineering, Mayo Clinic, Scottsdale, AZ 85259, USA

^3^ First Central Hospital of Tianjin Medical University, Tianjin 300070, China

^4^ School of Medicine, Nankai University, Tianjin 300071, China

^*^ These authors contributed equally to this work.

^#^ Corresponding author.

*E-mail addresses:* [Li.Zhuqing@mayo.edu](mailto:Li.Zhuqing@mayo.edu) (Z. Li), sxqmed@163.com (X. Sun), [kent1997wyx@163.com](mailto:kent1997wyx@163.com) (Y. Wang), [fengzhang0222@163.com](mailto:fengzhang0222@163.com) (F. Zhang), [wangliquniy@126.com](mailto:wangliquniy@126.com) (L. Wang), [Ai.Chunbo@mayo.edu](mailto:Ai.Chunbo@mayo.edu) (C. Ai), [yinxuemei0214@163.com](mailto:yinxuemei0214@163.com) (X. Yin), [lcl199405@163.com](mailto:lcl199405@163.com) (C. Liu), [874433965@qq.com](mailto:874433965@qq.com) (C. Li), [5020200072@nankai.edu.cn](mailto:5020200072@nankai.edu.cn) (C. Lu)

**Table of contents**

[Fig. S1 Supplementary physiological, echocardiographic, and tissue readouts supporting the multi-hit HFpEF model 3](#_Toc11442)

[Fig. S2 Supplementary transcriptomic and integrated proteomic–transcriptomic analyses for HFpEF 4](#_Toc2555)

[Fig. S3 Additional sympathetic markers and structural parameters following renal denervation in HFpEF mice 6](#_Toc19634)

[Fig. S4 Additional oxidative stress and cell injury parameters after renal denervation in HFpEF mice 6](#_Toc12232)

[Fig. S5 Transcriptomic profiling of neonatal rat ventricular myocytes confirms ATP-dependent inflammatory activation and its attenuation by P2X7 blockade 7](#_Toc5127)

[Fig. S6 8](#_Toc20789)

[Fig. S7. The whole uncropped images of the original Western blot in figure 5 9](#_Toc25755)

[Fig. S8. The whole uncropped images of the original Western blot in figure 6 10](#_Toc28550)

[Table S1. Primer Sequences and Product Sizes 11](#_Toc19868)

[Table S2. Compositions of normal chow and high-fat diets. 11](#_Toc3144)

[Table S3. Tail-cuff Blood Pressure at Baseline and Endpoint in Study Groups (mmHg). 12](#_Toc1325)

[Table S4. Comprehensive allocation matrix for all animal experiments 13](#_Toc31917)

**Supplemental figures**

**
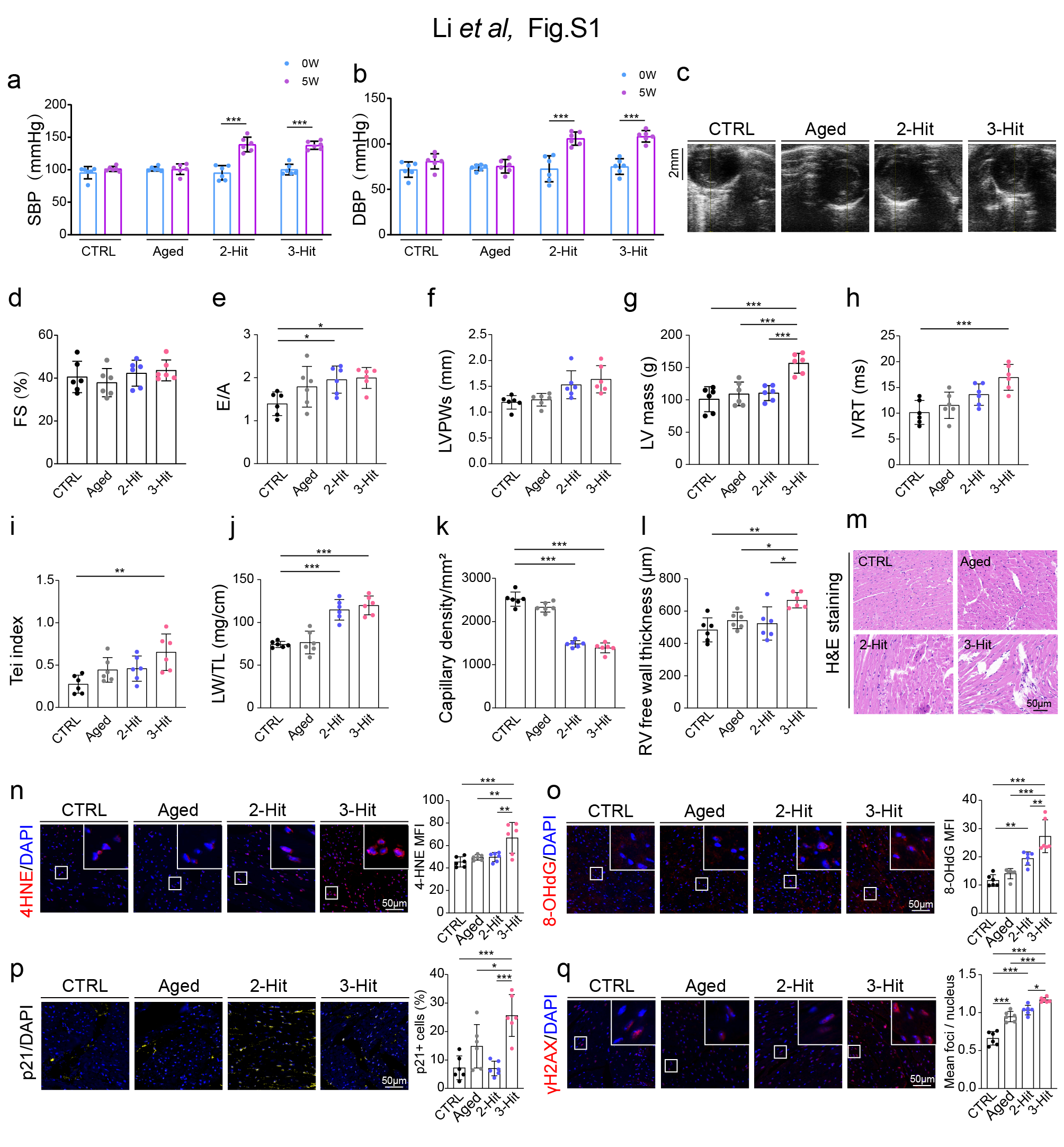
**

Fig. S1 Supplementary physiological, echocardiographic, and tissue readouts supporting the multi-hit HFpEF model. **a,b** Systolic (SBP) and diastolic (DBP) blood pressures at baseline (0 w, blue) and at 5 weeks (5 w, magenta) in CTRL, Aged, 2-Hit, and 3-Hit groups. **c** Representative parasternal short-axis M-mode echocardiograms for each group. **d** fractional shortening (FS); **e** early-to-late transmitral inflow ratio (E/A); **f** left-ventricular posterior-wall thickness in systole (LVPWs); **g** left-ventricular (LV) mass; **h** isovolumic relaxation time (IVRT); **i** myocardial performance (Tei) index = (ICT + IVRT)/ET, where ICT is isovolumic contraction time and ET is ejection time; **j** lung weight normalized to tibia length (LW/TL; mg/cm); **k** capillary density from CD31-stained sections; **l** right-ventricular (RV) free-wall thickness. **m** Representative H&E staining of LV myocardium. **n-q** Myocardial oxidative-stress and DNA-damage readouts in LV sections: **n** 4-hydroxynonenal (4-HNE) immunofluorescence with mean fluorescence intensity (MFI); **o** 8-hydroxy-2'-deoxyguanosine (8-OHdG) immunofluorescence with MFI; **p** p21 immunostaining with percentage of p21⁺ nuclei; q γH2AX foci per nucleus. Nuclei are counterstained with DAPI. Two-tailed unpaired Student’s t-test was performed for statistical analysis **(a,b)**. One-way ANOVA with Tukey’s post hoc was performed for statistical analysis (d-l, and n-q). n=6 mice/group. Data is presented as mean±SD. **P* < 0.05, ***P* < 0.01, ****P* < 0.001**
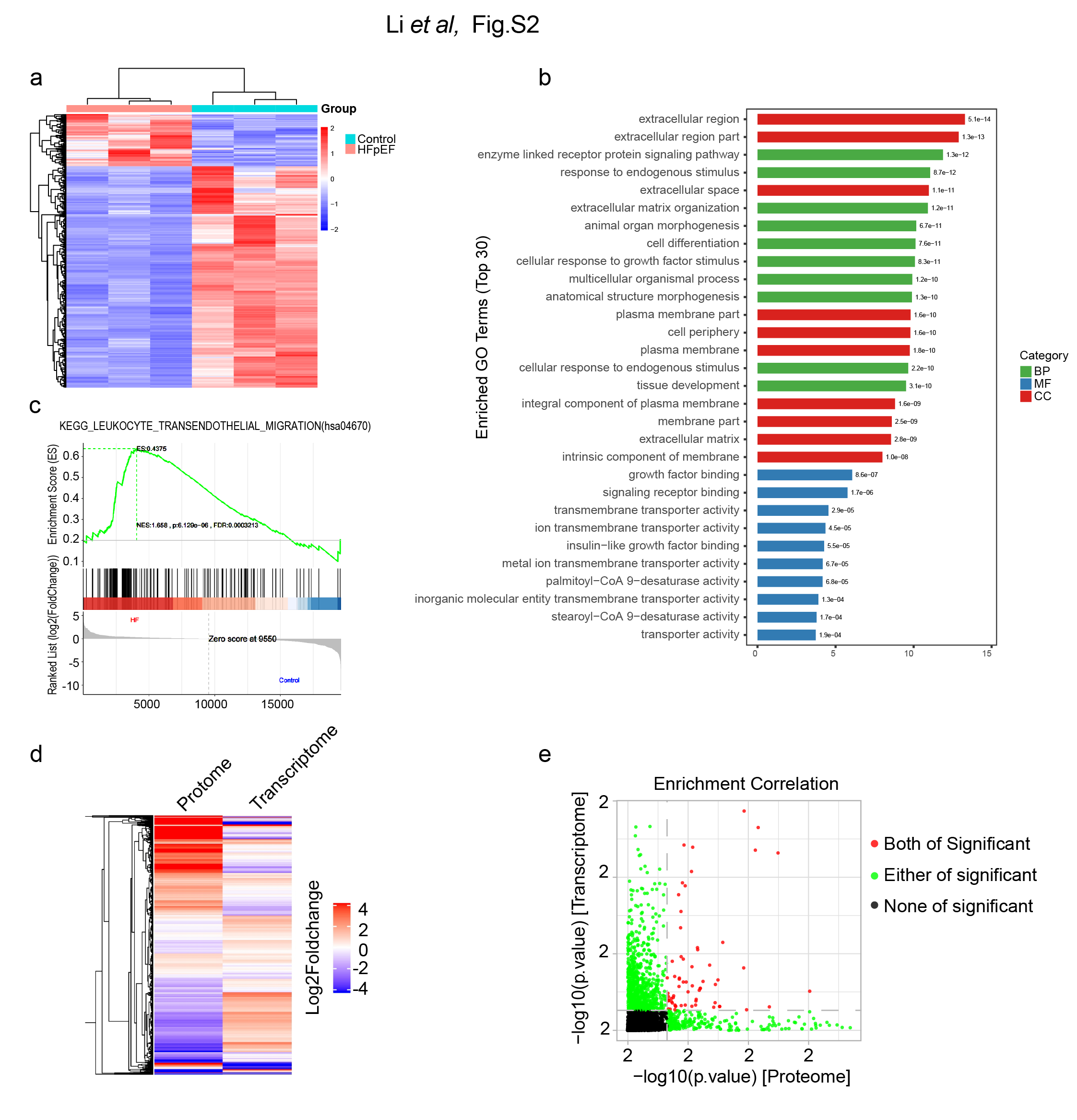
Fig. S2** Supplementary transcriptomic and integrated proteomic–transcriptomic analyses for HFpEF. **a** Hierarchical clustering heatmap of differentially expressed genes (DEGs) in HFpEF vs. Control. The color scale (blue to red) indicates lower to higher expression levels. **b** Top 30 significantly enriched Gene Ontology (GO) terms in the transcriptome, color-coded for Biological Process (BP), Molecular Function (MF), and Cellular Component (CC). The x-axis denotes -log10(p-value). **c** GSEA enrichment plot for KEGG Leukocyte Transendothelial Migration (hsa04670). The green curve represents the running enrichment score (ES), with NES, p-value, and FDR shown. **d** Combined heatmap comparing log2 (Fold Change) from the proteome (left) and transcriptome (right) for overlapping differentially expressed features, ranging from -4 (blue) to +4 (red). **e** Scatter plot correlating enrichment significance (-log10(p-value)) between the proteomic (x-axis) and transcriptomic (y-axis) analyses. Red dots denote terms significant in both datasets, green dots significant in either, and black dots not significant in either. Data support the involvement of immune and extracellular matrix pathways in HFpEF at both the mRNA and protein levels
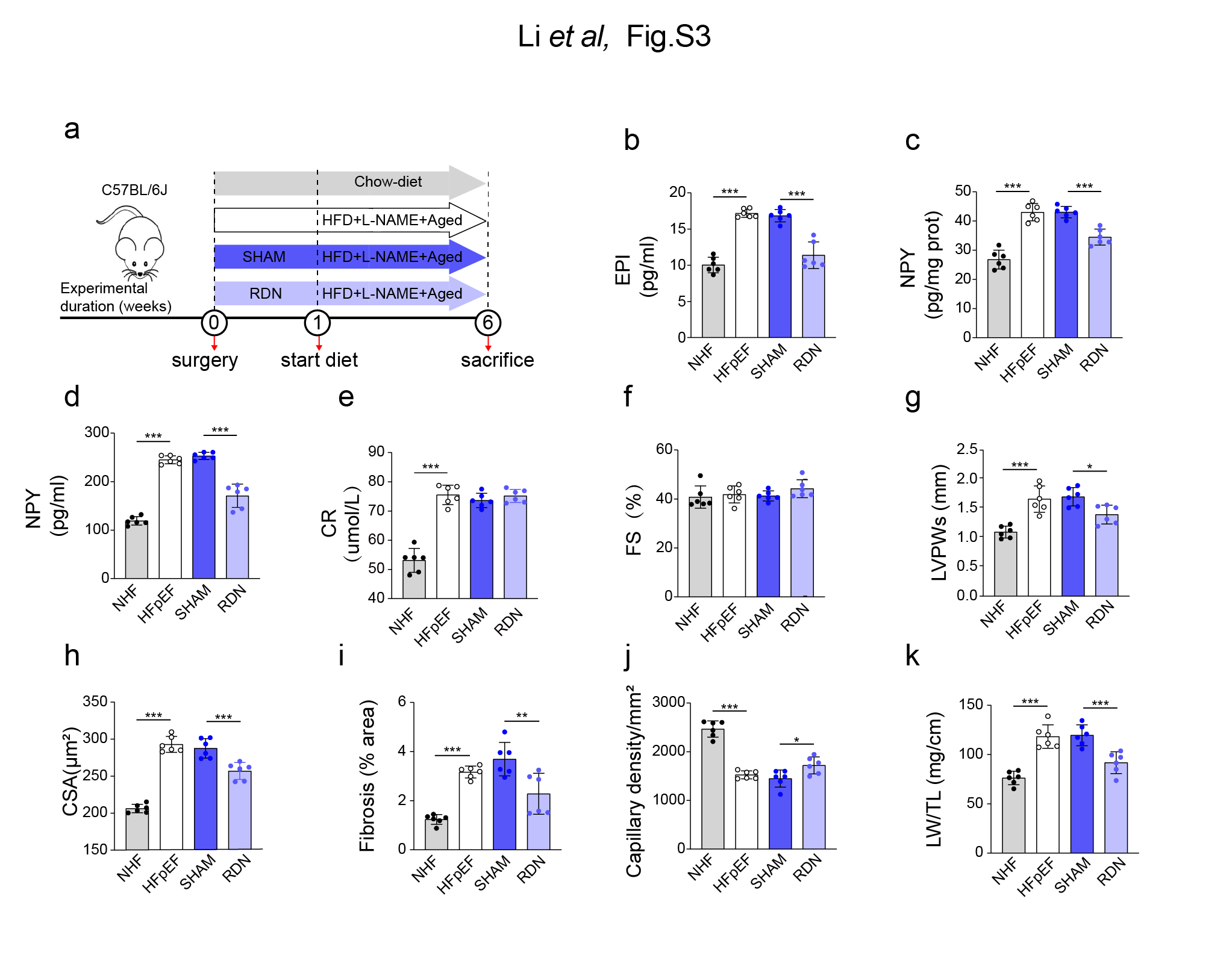


# **Fig. S3** Additional sympathetic markers and structural parameters following renal denervation in HFpEF mice. **b** Serum epinephrine (EPI). **c** Neuropeptide Y (NPY) in heart lysates. **d** Serum NPY. **e** Serum creatinine (CR). **f** Fractional shortening (FS). **g** Left ventricular posterior wall thickness in systole (LVPWs). **h** Cross-sectional area (CSA) of cardiomyocytes from WGA staining. **i** Myocardial fibrosis percentage. **j** Capillary density measured after CD31 staining. **k** Lung weight normalized to tibia length (LW/TL). One-way ANOVA with Tukey’s post hoc was performed for statistical analysis. n=6 mice/group. Data is presented as mean ± SD. **P* < 0.05; ***P* < 0.01; ****P* < 0.001


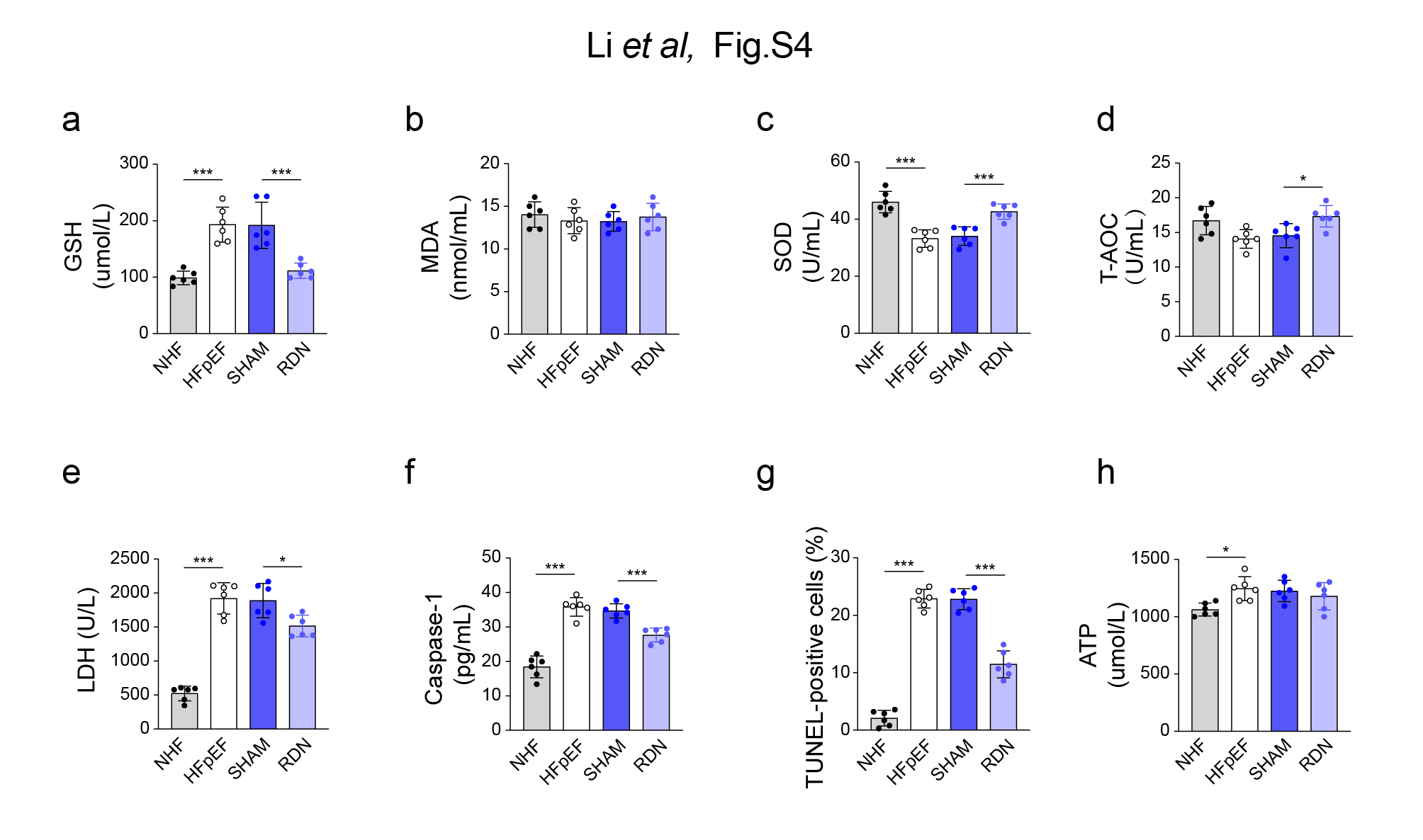


# **Fig. S4** Additional oxidative stress and cell injury parameters after renal denervation in HFpEF mice. **a** Serum glutathione (GSH). **b** Serum malondialdehyde (MDA). **c** Serum superoxide dismutase (SOD). **d** Serum total antioxidant capacity (T-AOC). **e** Serum lactate dehydrogenase (LDH). **f** Caspase-1 in heart lysates. **g** Percentage of TUNEL-positive cardiomyocytes in myocardial sections. **h** Serum ATP. Data is presented as mean ± SD. n=6 mice/group. **P* < 0.05; ***P* < 0.01; ****P*< 0.001


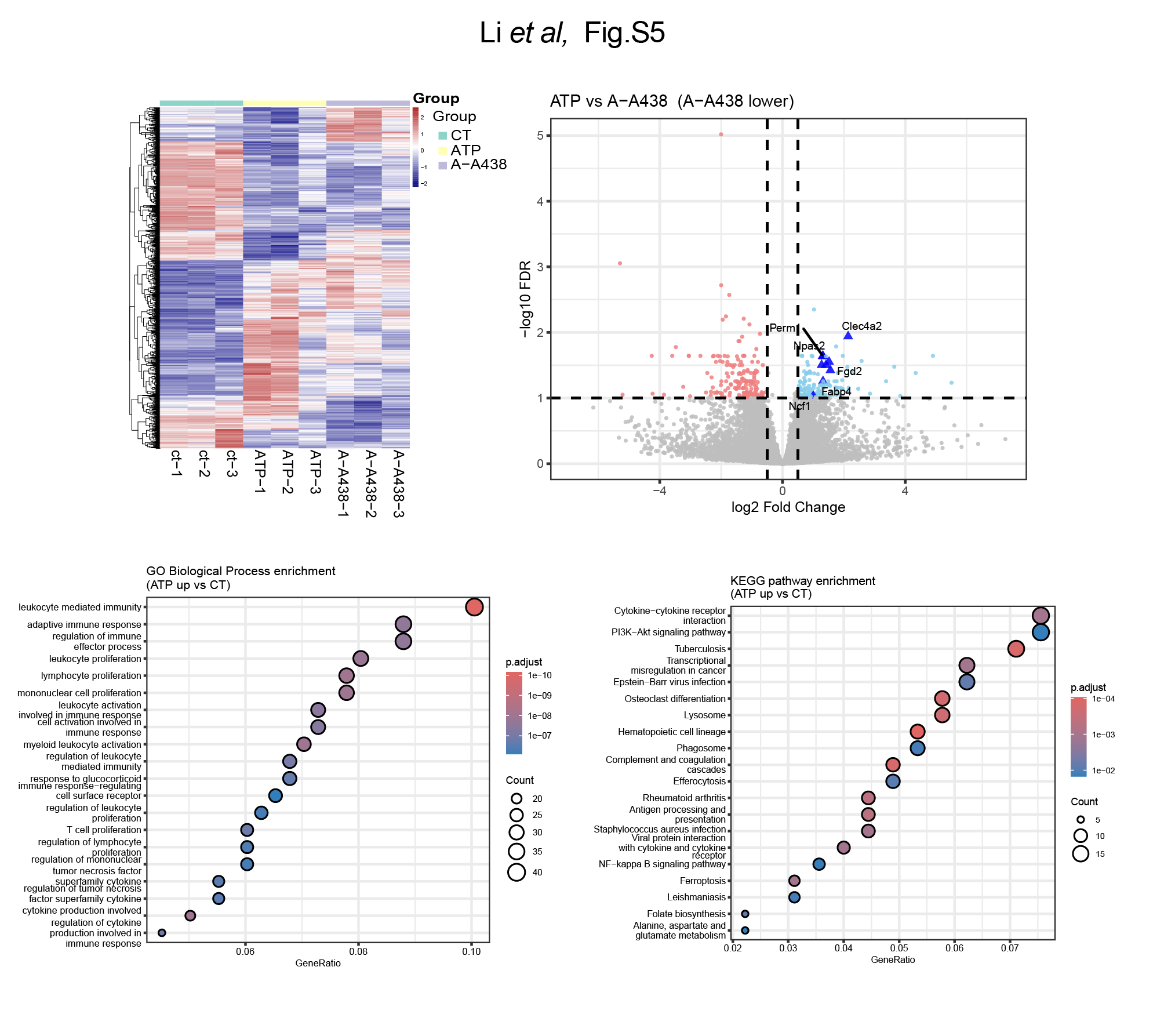
**Fig. S5** Transcriptomic profiling of neonatal rat ventricular myocytes confirms ATP-dependent inflammatory activation and its attenuation by P2X7 blockade. **a** Heat map of normalized expression across Control, ATP and ATP plus A438079 groups (n = 3 per group). Rows are genes and columns are samples. **b** Volcano plot for ATP versus ATP plus A438079. Positive log2 fold change indicates higher expression in ATP. Selected genes related to inflammatory signaling are labeled. **c** Gene Ontology Biological Process enrichment for genes upregulated by ATP versus Control. Enriched terms include leukocyte mediated immunity, regulation of immune effector process, leukocyte and lymphocyte proliferation, leukocyte activation and cytokine production involved in immune response. **d** KEGG pathway enrichment for genes upregulated by ATP versus Control. Representative pathways include cytokine cytokine receptor interaction, PI3K Akt signaling, hematopoietic cell lineage, complement and coagulation cascades, antigen processing and presentation, phagosome, lysosome, ferroptosis and NF kappa B signaling. Differential expression was assessed with FDR adjusted *P* values using Benjamini–Hochberg correction. Bubble plots display gene ratio on the x axis, adjusted *P* by color and gene count by point size.

**
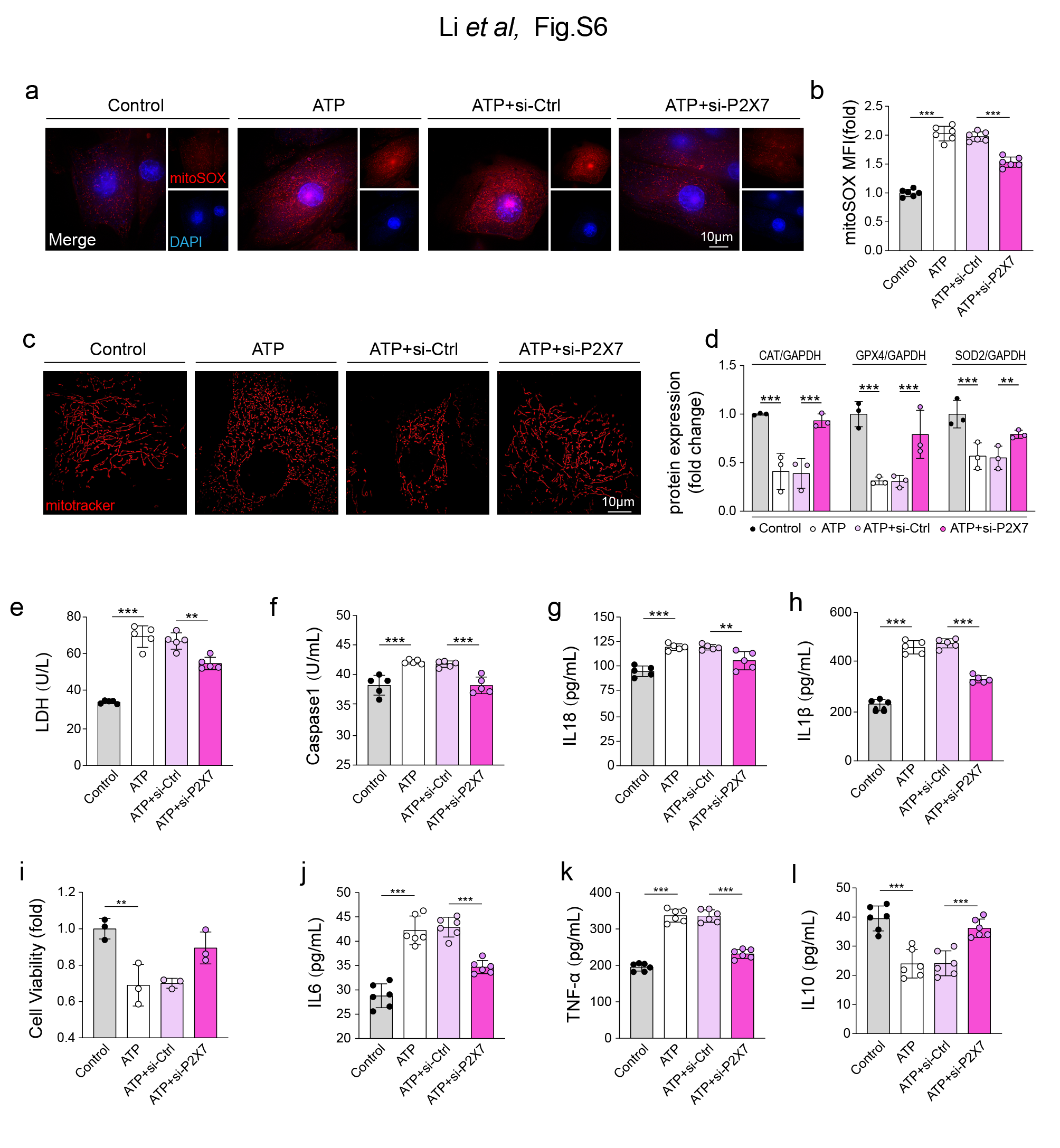
**

Fig. S6 P2X7 knockdown attenuates ATP-induced mitochondrial ROS, oxidative stress, and pyroptosis in H9c2 cardiomyocytes. H9c2 cells were transfected with control siRNA (si-Ctrl) or P2X7 siRNA (si-P2X7) for 24 h and then exposed to ATP (5 mM) for a further 24 h. **a** Representative MitoSOX images of mitochondrial superoxide (red) with DAPI (blue). **b** Quantification of MitoSOX MFI (fold) (n=6). **c** MitoTracker staining showing mitochondrial network morphology. **d** Quantification of Western blots of key antioxidant enzymes (CAT, GPX4, SOD2) in H9c2 cells under siRNA treatments (n = 3). **g** Lactate dehydrogenase (LDH) activity in supernatants (n=5). **h** Caspase-1 activity (n=5). **i, j** Pro-inflammatory cytokines in supernatants: IL-18 **(i)** and IL-1β **(j)** (n=5). **k** Cell viability by CCK-8 (fold to Control) (n=3). **l-n** Additional cytokines in supernatants: IL-6 **(l)**, TNF-α **(m)**, and IL-10 **(n)** (n=6). One-way ANOVA with Tukey’s post hoc was performed for statistical analysis. Data is presented as mean ± SD. **P* < 0.05; ***P* < 0.01; ****P* < 0.001

# Fig. S7. The whole uncropped images of the original Western blot in figure 5

**
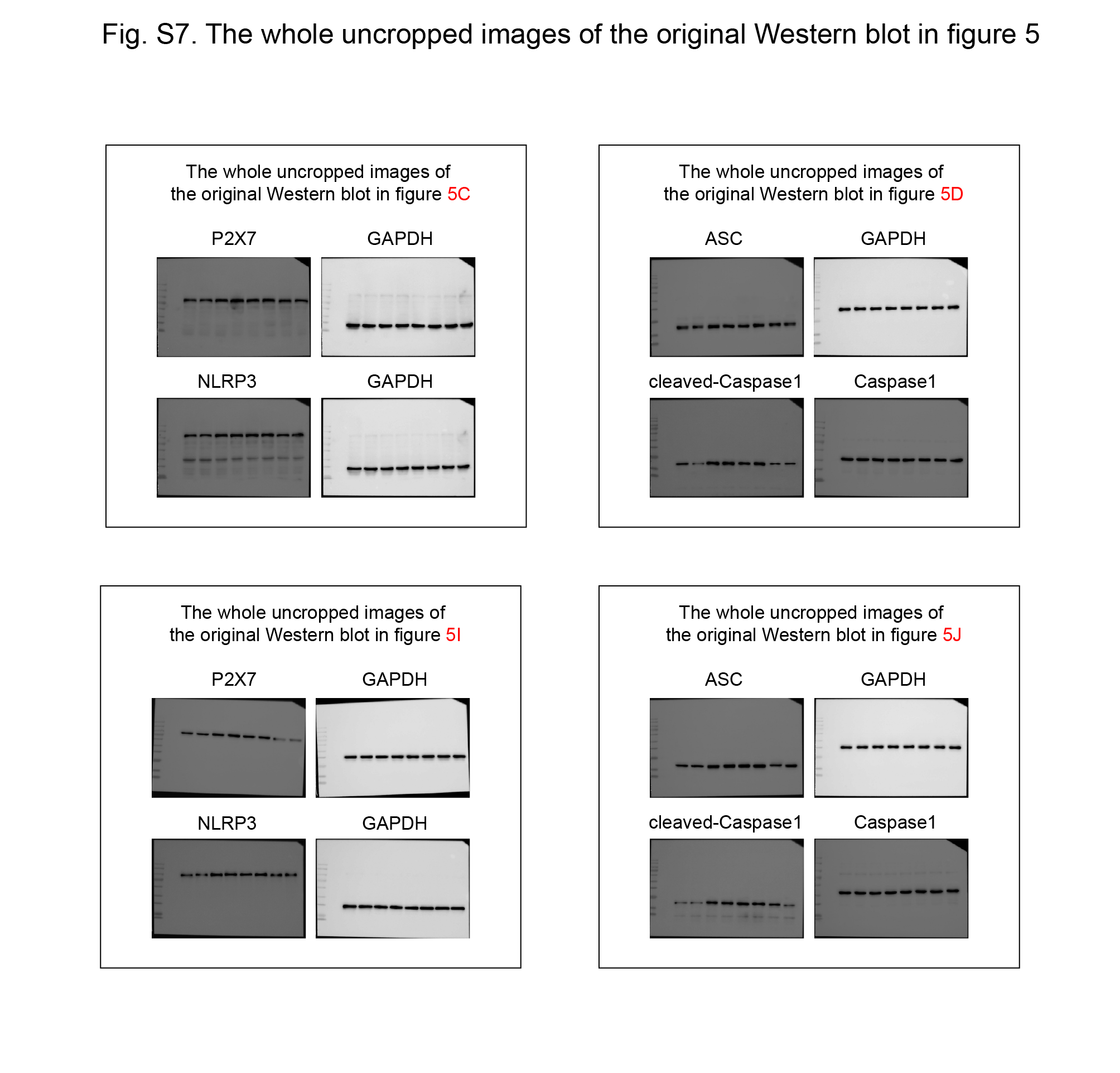
**

# Fig. S8. The whole uncropped images of the original Western blot in figure 6


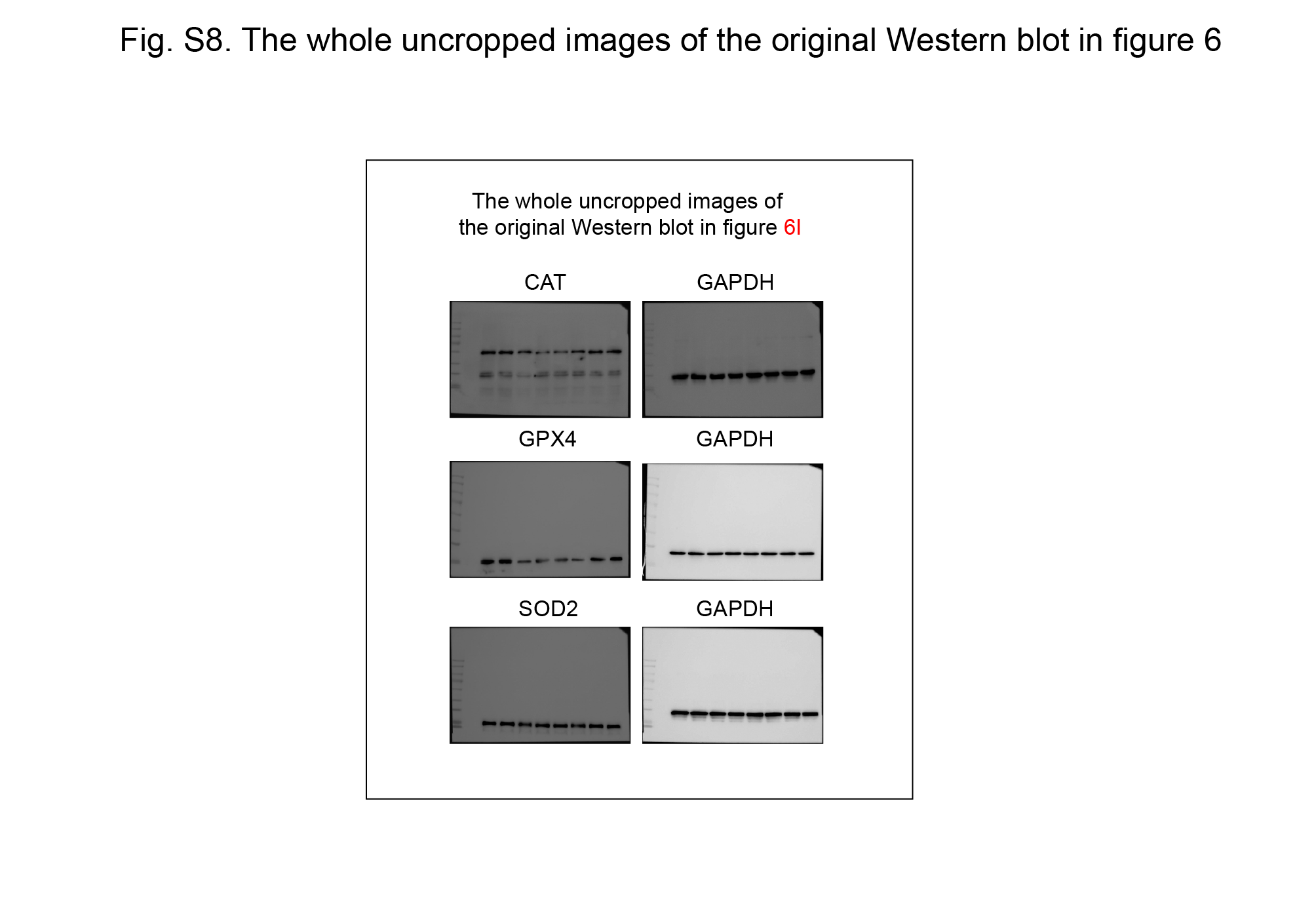


**Supplemental tables**

# Table S1. Primer Sequences and Product Sizes

| Primer name |  | Sequence（5’-3’） | Size（bp） |
| --- | --- | --- | --- |
| *Cola2* | F | CCCAAGGACTATGAAGTTGATGC | 155 |
|  | R | TCAATCCAGTAGTAATCGCTGTTCC |  |
| *Timp1* | F | CAGTGTTTCCCTGTTTATCTATCCC | 111 |
|  | R | AAGCAAAGTGACGGCTCTGGTAG |  |
| *Fn1* | F | GCCCTACCAAGGCTGGATGATG | 160 |
|  | R | AAGCAGGTTTCCTCGGTTGTCC |  |
| *α-SMA* | F | ATCAGGGAGTAATGGTTGGAATGG | 204 |
|  | R | CCTCTGTCAGCAGTGTCGGATG |  |
| *GAPDH* | F | CGGTGCTGAGTATGTCGTGGAGTC | 100 |
|  | R | GGCGGAGATGATGACCCTTTTG |  |

# Table S2. Compositions of normal chow and high-fat diets.

| Components | Normal Chow | High-Fat Diet |
| --- | --- | --- |
| Crude protein (%) | 19.24 | 26.2 |
| Crude fat (%) | 4.26 | 34.9 |
| Crude fiber (%) | 4.73 | 6.5 |
| Crude ash (%) | 1.0 | 1.0 |
| Moisture (%) | 8.9 | 8.9 |
| Calcium (%) | 0.83 | 0.83 |
| Total phosphorus (%) | 0.69 | 0.69 |
| Energy (kcal/g) | 3.82 | 5.21 |
| Energy ratio | Protein 20%, Fat 10%, Carbohydrate 70% | Protein 20%, Fat 60%, Carbohydrate 20% |

# Table S3. Tail-cuff Blood Pressure at Baseline and Endpoint in Study Groups (mmHg).

| group | SBP Baseline | SBP Endpoint | DBP Baseline | DBP Endpoint |
| --- | --- | --- | --- | --- |
| NHF | 95.87 ± 3.36 | 101.33 ± 1.54 | 69.64 ± 2.48 | 73.50 ± 1.03 |
| HFpEF | 103.61 ± 3.16 | 136.83 ± 1.42 | 74.00 ± 3.14 | 109.50 ± 2.17 |
| SHAM | 101.83 ± 2.41 | 139.00 ± 1.37 | 76.83 ± 2.43 | 106.50 ± 2.63 |
| RDN | 104.17 ± 2.20 | 123.40 ± 2.31* | 74.56 ± 3.43 | 93.44 ± 1.31* |

Data are mean ± SEM (n = 6 per group). **P* < 0.05 versus SHAM at the corresponding time point (two-sided test).
Abbreviations: SBP, systolic blood pressure; DBP, diastolic blood pressure.

# Supplementary Table S4. Comprehensive allocation matrix for all animal experiments

Section A-Experiment 1: CTRL / AGED / 2-Hit / 3-Hit (Figs 1–2; Suppl. Fig. S1)

| Mouse ID | Echo (Fig.1 & Suppl. Fig. 1) | Histology (Fig. 1 & Suppl. Fig. 1) | Molecular (ELISA) (Fig. 1 & Suppl. Fig. 1) | Molecular (qPCR) (Fig. 1) | TEM (Fig. 1) | Blood pressure, body weight, glucose (Fig. 1 & Suppl. Fig. 1) | Omics sequencing (Fig. 2 & Suppl. Fig. 2) | Exercise tolerance, HW/TL, LW/TL (Fig. 1 & Suppl. Fig. 1) |
| --- | --- | --- | --- | --- | --- | --- | --- | --- |
| CTRL-1 | √ | √ | √ | √ | √ | √ | × | √ |
| CTRL-2 | √ | √ | √ | √ | √ | √ | × | √ |
| CTRL-3 | √ | √ | √ | √ | × | √ | √ | √ |
| CTRL-4 | √ | √ | √ | √ | √ | √ | × | √ |
| CTRL-5 | √ | √ | √ | × | × | √ | √ | √ |
| CTRL-6 | √ | √ | √ | √ | × | √ | √ | √ |
| Aged-1 | √ | √ | √ | √ | √ | √ | × | √ |
| Aged-2 | √ | √ | √ | √ | × | √ | √ | √ |
| Aged-3 | √ | √ | √ | √ | √ | √ | × | √ |
| Aged-4 | √ | √ | √ | √ | × | √ | √ | √ |
| Aged-5 | √ | √ | √ | × | √ | √ | × | √ |
| Aged-6 | √ | √ | √ | √ | × | √ | √ | √ |
| 2-Hit-1 | √ | √ | √ | √ | √ | √ | × | √ |
| 2-Hit-2 | √ | √ | √ | √ | √ | √ | × | √ |
| 2-Hit-3 | √ | √ | √ | √ | × | √ | √ | √ |
| 2-Hit-4 | √ | √ | √ | √ | × | √ | √ | √ |
| 2-Hit-5 | √ | √ | √ | × | √ | √ | × | √ |
| 2-Hit-6 | √ | √ | √ | √ | × | √ | √ | √ |
| 3-Hit-1 | √ | √ | √ | √ | √ | √ | × | √ |
| 3-Hit-2 | √ | √ | √ | √ | × | √ | √ | √ |
| 3-Hit-3 | √ | √ | √ | √ | × | √ | √ | √ |
| 3-Hit-4 | √ | √ | √ | √ | √ | √ | × | √ |
| 3-Hit-5 | √ | √ | √ | × | √ | √ | × | √ |
| 3-Hit-6 | √ | √ | √ | √ | × | √ | √ | √ |

Section B-Experiment 2: RDN intervention (Figs 3–5; Suppl. Figs S3–S4)

| Mouse ID | Echo (Fig.3 & Suppl. Fig. 3) | Histology (Fig. 3-5 & Suppl. Fig. 3) | Molecular (ELISA) (Fig.3-5 & Suppl. Fig. 3-4) | Molecular (WB) (Fig.5) | Molecular (qPCR) (Fig.3) | TEM (Fig.4) | Blood pressure | TUNEL (Fig.4 & Suppl. Fig. 4) | Exercise tolerance, HW/TL, LW/TL (Fig. 3 & Suppl. Fig. 3) |
| --- | --- | --- | --- | --- | --- | --- | --- | --- | --- |
| NHF-1 | √ | √ | √ | × | √ | √ | √ | √ | √ |
| NHF-2 | √ | √ | √ | √ | √ | × | √ | √ | √ |
| NHF-3 | √ | √ | √ | √ | √ | × | √ | √ | √ |
| NHF-4 | √ | √ | √ | × | √ | √ | √ | √ | √ |
| NHF-5 | √ | √ | √ | × | √ | √ | √ | √ | √ |
| NHF-6 | √ | √ | √ | √ | × | × | √ | √ | √ |
| HFpEF-1 | √ | √ | √ | √ | √ | × | √ | √ | √ |
| HFpEF-2 | √ | √ | √ | × | √ | √ | √ | √ | √ |
| HFpEF-3 | √ | √ | √ | × | √ | √ | √ | √ | √ |
| HFpEF-4 | √ | √ | √ | × | √ | √ | √ | √ | √ |
| HFpEF-5 | √ | √ | √ | √ | × | × | √ | √ | √ |
| HFpEF-6 | √ | √ | √ | √ | √ | × | √ | √ | √ |
| Sham-1 | √ | √ | √ | √ | × | × | √ | √ | √ |
| Sham-2 | √ | √ | √ | √ | √ | × | √ | √ | √ |
| Sham-3 | √ | √ | √ | × | √ | √ | √ | √ | √ |
| Sham-4 | √ | √ | √ | × | √ | √ | √ | √ | √ |
| Sham-5 | √ | √ | √ | √ | √ | × | √ | √ | √ |
| Sham-6 | √ | √ | √ | × | √ | √ | √ | √ | √ |
| RDN-1 | √ | √ | √ | √ | × | × | √ | √ | √ |
| RDN-2 | √ | √ | √ | × | √ | √ | √ | √ | √ |
| RDN-3 | √ | √ | √ | √ | √ | × | √ | √ | √ |
| RDN-4 | √ | √ | √ | × | √ | √ | √ | √ | √ |
| RDN-5 | √ | √ | √ | √ | √ | × | √ | √ | √ |
| RDN-6 | √ | √ | √ | × | √ | √ | √ | √ | √ |

Section C-Experiment 3: A438079 intervention (Figure 7)

| Mouse ID | Echo (Fig.7) | Histology (Fig.7) | Molecular (ELISA) (Fig.7) | Molecular (qPCR) (Fig.7) | Exercise tolerance, HW/TL (Fig. 7) |
| --- | --- | --- | --- | --- | --- |
| NHF-Vehicle-1 | √ | √ | √ | √ | √ |
| NHF-Vehicle-2 | √ | √ | √ | √ | √ |
| NHF-Vehicle-3 | √ | √ | √ | √ | √ |
| NHF-Vehicle-4 | √ | √ | √ | √ | √ |
| NHF-Vehicle-5 | √ | √ | √ | √ | √ |
| NHF-Vehicle-6 | √ | √ | √ | × | √ |
| NHF-A438079-1 | √ | √ | √ | √ | √ |
| NHF-A438079-2 | √ | √ | √ | √ | √ |
| NHF-A438079-3 | √ | √ | √ | √ | √ |
| NHF-A438079-4 | √ | √ | √ | √ | √ |
| NHF-A438079-5 | √ | √ | √ | √ | √ |
| NHF-A438079-6 | √ | √ | √ | × | √ |
| HFpEF-Vehicle-1 | √ | √ | √ | √ | √ |
| HFpEF-Vehicle-2 | √ | √ | √ | √ | √ |
| HFpEF-Vehicle-3 | √ | √ | √ | √ | √ |
| HFpEF-Vehicle-4 | √ | √ | √ | √ | √ |
| HFpEF-Vehicle-5 | √ | √ | √ | √ | √ |
| HFpEF-Vehicle-6 | √ | √ | √ | × | √ |
| HFpEF-A438079-1 | √ | √ | √ | √ | √ |
| HFpEF-A438079-2 | √ | √ | √ | √ | √ |
| HFpEF-A438079-3 | √ | √ | √ | √ | √ |
| HFpEF-A438079-4 | √ | √ | √ | √ | √ |
| HFpEF-A438079-5 | √ | √ | √ | √ | √ |
| HFpEF-A438079-6 | √ | √ | √ | × | √ |

Section D-Experiment 3: MCC950 intervention (Figure 8)

| Mouse ID | Echo (Fig.8) | Histology (Fig.8) | Molecular (qPCR) (Fig.8) | Molecular (ELISA) (Fig.8) | Exercise tolerance, HW/TL (Fig. 8) |
| --- | --- | --- | --- | --- | --- |
| NHF-VEH-1 | √ | √ | √ | √ | √ |
| NHF-VEH-2 | √ | √ | √ | √ | √ |
| NHF-VEH-3 | √ | √ | √ | √ | √ |
| NHF-VEH-4 | √ | √ | √ | √ | √ |
| NHF-VEH-5 | √ | √ | √ | √ | √ |
| NHF-VEH-6 | √ | √ | √ | × | √ |
| NHF-MCC950-1 | √ | √ | √ | √ | √ |
| NHF-MCC950-2 | √ | √ | √ | √ | √ |
| NHF-MCC950-3 | √ | √ | √ | √ | √ |
| NHF-MCC950-4 | √ | √ | √ | √ | √ |
| NHF-MCC950-5 | √ | √ | √ | √ | √ |
| NHF-MCC950-6 | √ | √ | √ | × | √ |
| HFpEF-VEH-1 | √ | √ | √ | √ | √ |
| HFpEF-VEH-2 | √ | √ | √ | √ | √ |
| HFpEF-VEH-3 | √ | √ | √ | √ | √ |
| HFpEF-VEH-4 | √ | √ | √ | √ | √ |
| HFpEF-VEH-5 | √ | √ | √ | √ | √ |
| HFpEF-VEH-6 | √ | √ | √ | × | √ |
| HFpEF-MCC950-1 | √ | √ | √ | √ | √ |
| HFpEF-MCC950-2 | √ | √ | √ | √ | √ |
| HFpEF-MCC950-3 | √ | √ | √ | √ | √ |
| HFpEF-MCC950-4 | √ | √ | √ | √ | √ |
| HFpEF-MCC950-5 | √ | √ | √ | √ | √ |
| HFpEF-MCC950-6 | √ | √ | √ | × | √ |
